# Dementia care pathways in prisons – a comprehensive scoping review

**DOI:** 10.1186/s40352-023-00252-7

**Published:** 2024-01-20

**Authors:** Samantha Treacy, Steven Martin, Nelum Samarutilake, Veronica Phillips, Ben R. Underwood, Tine Van Bortel

**Affiliations:** 1 Faculty of Humanities and Social Sciences, Department of Criminology, Sociology & Social Policy, Swansea University, Swansea, UK; 2 Leicester School of Allied Health Sciences, De Montfort University, Leicester, UK; 3 Department of Psychiatry, School of Clinical Medicine, University of Cambridge, Cambridge, UK; 4 Cambridge Medical Library, School of Clinical Medicine, University of Cambridge, Cambridge, UK; 5 Cambridgeshire and Peterborough NHS Foundation Trust, Cambridge, UK

**Keywords:** Prisons, People living in prison, Dementia, Care pathways

## Abstract

**Background:**

The number of older people in prison is growing. As a result, there will also be more prisoners suffering from dementia. The support and management of this population is likely to present multiple challenges to the prison system.

**Objectives:**

To examine the published literature on the care and supervision of people living in prison with dementia and on transitioning into the community; to identify good practice and recommendations that might inform the development of prison dementia care pathways.

**Methods:**

A scoping review methodology was adopted with reporting guided by the PRISMA extension for scoping reviews checklist and explanation.

**Results:**

Sixty-seven papers were included. Most of these were from high income countries, with the majority from the United Kingdom (*n* = 34), followed by the United States (*n* = 15), and Australia (*n* = 12). One further paper was from India.

**Discussion:**

The literature indicated that there were difficulties across the prison system for people with dementia along the pathway from reception to release and resettlement. These touched upon all aspects of prison life and its environment, including health and social care. A lack of resources and national and regional policies were identified as important barriers, although a number of solutions were also identified in the literature, including the development of locally tailored policies and increased collaboration with the voluntary sector.

**Conclusion:**

To our knowledge, this is the most comprehensive and inclusive review of the literature on dementia care pathways in prison to date. It has identified a number of important areas of concern and opportunities for future research across the prison system, and its operations. This will hopefully lead to the identification or adaptation of interventions to be implemented and evaluated, and facilitate the development of dementia care pathways in prisons.

**Supplementary Information:**

The online version contains supplementary material available at 10.1186/s40352-023-00252-7.

## Background

The number of older people (defined here as those over 50^1^) being held in prison in England and Wales has almost tripled over the last 20 years, and they now represent 17.1% of that population (Ministry of Justice, 2022a). The growing number of older people has brought with it an increasing number of health and social care problems, reportedly affecting around 85% of older people in prison, with associated costs (Di Lorito, et al., 2018; Hayes et al., 2012, 2013; Senior, et al., 2013). It has been estimated that 8.1% of those over the age of 50 in prison have mild cognitive impairment or dementia, which is much higher than estimates for this age group in the general population (Dunne et al., 2021; Forsyth et al., 2020). This pattern of poor health also increased the vulnerability of older people in prison during the pandemic (Kay, 2020).

Prison policy and legislation mandates that health and social care be ‘equivalent’ to that provided in the community (Care Act, 2014; Department of Health, 1999). Despite this, provisions are reportedly inconsistent, and the government has been described as ‘failing’ in its duty of care (Health and Social Care Committee, 2018; HM Inspectorate of Prisons & Care Quality Commission, 2018). This is likely exacerbated by the suspension and limiting of healthcare services during the pandemic, noted to have had a ‘profound’ impact on people’s health and wellbeing (HM Inspectorate of Prisons, 2021). This may be particularly so for people living in prison with dementia (PLiPWD), whereby the difficulties of delivering health and social care are compounded by inappropriate buildings, environments, and prison regimes (rules and regulations). In addition, PLiPWDs may experience an increase in social isolation, including separation from friends and family, all of which may make their time in prison more challenging (Moll, 2013; Peacock et al., 2019).

There is no current national strategy for older people in prison in England and Wales, including PLiPWD, although the British government recently agreed that there is a need for one (Justice Committee, 2020). A ‘Model for Operational Delivery’ for older people has been published by Her Majesty's Prison & Probation Service (2018) in England and Wales, though this is guidance only and the “properly resourced and coordinated strategy” previously called for has not been produced (Prisons & Probation Ombudsman, 2017, p7; Brooke and Rybacka, 2020; HM Inspectorate of Prisons, 2019; Justice Committee, 2020). One way of attempting to standardise and improve the quality of treatment and care in the community has been through the use of care pathways (Centre for Policy on Ageing, 2014; Schrijvers et al., 2012). Care pathways have been defined as “a complex intervention for the mutual decision-making and organisation of care processes for a well-defined group of patients during a well-defined period”, involving an articulation of goals and key aspects of evidence-based care, coordination and sequencing of activities and outcomes evaluation (Vanhaecht, et al., 2007, p137).

The development of care pathways within the prison system lags behind that of the community, but the National Institute for Health and Care Excellence (NICE) has produced a pathway for prisoner health for England and Wales (National Institute for Health and Care Excellence, 2019), and there is a care pathway for older prisoners in Wales (Welsh Government & Ministry of Justice, 2011). There has also recently been an overall care pathway developed for people in prison with mild cognitive impairment and dementia, although this has not been implemented as yet, and it does not include any details regarding release and resettlement (Forsyth et al, 2020). It has been recommended that care pathways should be developed locally, as they are context-sensitive, should be viewed as processual and flexible, and the needs of the person, their experiences and characteristics need to be taken into account – such as age, gender and race (Centre for Policy on Ageing, 2014; Pinder, et al., 2005).

Here we review the current literature on people living in prison with dementia. There have been two recent systematic literature reviews conducted on PLiPWD, both of which only included primary research studies that were small in number (Brooke and Rybacka, 2020 (*n* = 10); Peacock et al., 2019 (*n* = 8)), and focused on prevalence, identification (screening and diagnosis), and the need for tailored programming and staff training. Peacock et al., (2019) identified dementia as a concern and suggested recommendations for improved screening and care practices. Brooke et al. (2020) noted that, whilst the prevalence of dementia in prison populations was largely unknown, there was a need for national policies and local strategies that support a multi-disciplinary approach to early detection, screening and diagnosis. Neither paper, however, reported on the much more extensive and rich grey literature in this area (Brooke and Rybacka, 2020), to help comprehensively identify the systemic and operational problems, barriers and potential solutions that would be useful to consider in developing local dementia care pathways. Therefore, the aim of this paper is to conduct a comprehensive systematic scoping review of the available published literature on the support and management of PLiPWD in prison and upon transitioning into the community, and to identify practice and recommendations that would be useful to consider in the development of a local prison dementia care pathway.

## Methods

A scoping review methodology using Arksey and O’Malley’s (2005) five-stage framework was adopted for this review. Reporting was guided by the PRISMA extension for scoping reviews checklist and explanation (Tricco et al., 2018). The completed checklist for this review is available in Additional file 1: Appendix 1.

### Identification of relevant reports

The search strategy was formulated by the research team, and included an electronic database search and subsequent hand search. The electronic search involved searching twelve electronic databases: Applied Social Sciences Index and Abstract, Criminal Justice Abstracts, Embase, Medline (OVID), National Criminal Justice Reference Service, Open Grey, Psycinfo, Pubmed, Scopus, Social Services Abstracts, Sociological Abstracts, and Web of Science. The search combined condition-related terms (dementia OR Alzheimer*) AND context-related ones (prison OR jail OR gaol OR penitentia* OR penal OR correctional* OR incarcerat*), with no date or language restrictions, and covered the full range of publications up until April 2022. Additional file 2: Appendix 2 has an example of the search strategy used.

Electronic searches were supplemented by comprehensive hand searching and reference mining. Searches were also undertaken using: search engines; websites related to prisons and/or dementia (for example, Prison Reform Trust); a database from a previous related literature review (Lee et al, 2019); recommendations from academic networking sites; contacting prominent authors in the field directly; government-related websites (for example Public Health England, now called Health Security Agency); recent inspection reports for all prisons in England and Wales from Her Majesty’s Inspectorate of Prisons and the Independent Monitoring Board.

### Inclusion and exclusion criteria

Papers were considered suitable for inclusion in this review if they met the following criteria:

(i) Setting: Papers should primarily be set in, or pertain to, prisons. Documents solely referring to community services, hospitals or medical facilities that are not part of the prison system were excluded.
(ii) People: Papers involving PLiPWD. Research focused only on older people in prison more generally was excluded, as was research which described the disorienting effects of imprisonment more generally, but which was not related to dementia.
(iii) Intervention: Some consideration of the treatment, care, support or management of PLiPWD; this can be health or social-care associated, as well as related to the prison overall, and to any individuals, groups or agencies who visit or work with individuals during their time in prison (including family, friends, charities, probation services). Papers which mostly describe prevalence studies, sentencing practices or profiles were excluded.
(iv) Study design: All designs were considered for inclusion. Editorials, book reviews, online blogs, press releases, announcements, summaries, newspaper and magazine articles, abstracts and letters were excluded.

The titles, abstracts and full-text of the papers identified by the searches were screened for inclusion in the review. The screening was undertaken by two independent researchers (ST and NS) for inter-rater reliability purposes (Rutter et al., 2010). Any differences of opinion on inclusion were resolved between the researchers (ST, NS and SM), and with the Principle Investigator (TVB).

### Charting the data

An extraction template was developed for the review, guided by the PICO formula (Richardson et al., 1995) and informed by pathway stages and key areas highlighted in the older prisoner pathways toolkit for England and Wales (Department of Health, 2007), and the older prisoner pathway formulated for Wales (Welsh Government & Ministry of Justice, 2011). Using this extraction template, all of the data was extracted from the included papers by one member of the research team (ST), with a second researcher extracting data from a third of the papers as a check for consistency (SM). Any unresolved issues were related to the Principle Investigator (TVB) for resolution.

### Collating, summarising and reporting results

The review was deliberately inclusive of a wide variety of types of papers, which meant that taking a meta-analytic approach to the data was not feasible. Therefore, a narrative approach to summarising and synthesising the findings and recommendations of the included papers was adopted (Popay et al, 2006).

## Results

**Fig. 1:**
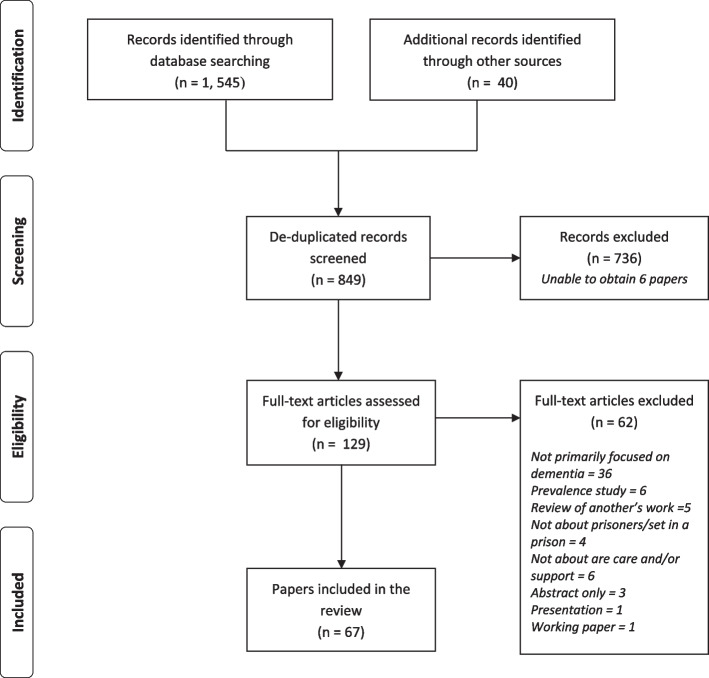
PRISMA flow diagram

Sixty-seven papers were included in this scoping review. The screening process phases conducted by the research team are shown in Fig. 1.

A brief overview of the key features of each of the papers is presented in Table 1. All but one of the included papers were from high income countries, with the majority from the United Kingdom (*n* = 34), and then the United States (*n* = 15), Australia (*n* = 12), Canada (*n* = 4), Italy (*n* = 1) and India (*n* = 1). The papers were split into types, with twenty-two guidance and inspection documents, and twenty-seven discussion and intervention description papers. Of the eighteen research and review articles with a defined methodology included there were four literature reviews (one was systematic), nine qualitative studies, four mixed-methods studies (one which followed participants up), and one survey-based study.

**Table 1:** Main features of the included papers

Study No	Author, Year, Country	Study type	Study Aims	Study Design	Sample size, type & setting	Intervention(s)	Main conclusion(s)
***(1) Research & review papers***							
1	Brooke et al, 2018, UK	Review	To identify how PLPWDs are cared for & supported	Systematic review	10 papers (UK = 3, USA = 3, Australia = 2, France = 1, Sweden = 1)	None reported	Need to find ways to identify need; lack of officer & legal professionals’ knowledge
2	Brooke & Jackson, 2019, UK	Qualitative	To understand staff & peer experiences of work with people with dementia	Interviews & focus groups	*n* = 29 (peer support = 5; MDT staff = 24). Male sex offender prison	n/a	Diversity in staff and peer supporters’ training & roles, and the prison regime. Need for training, and policy & guideline development
3	Brown, 2016, Australia	Qualitative	To investigate ‘effective’ programmes for prisoners with dementia	Interviews—staff, focus gp—prisoners	*n* = 24 (14 staff, 7 prisoners, 3 experts); 4 prisons: USA = 3, NZ = 1	Care programmes, peer support, environmental adaptations	Person-centredness, early identification, training, suitable care facilities in prison & community important, with policy needing to be developed
4	Cipriani et al., 2017, Italy	Review	To explore the ‘phenomenon’ of people with dementia in prison	Systematic search, quali-tative synthesis	50 papers	Unclear – papers not differentiated by intervention	Lack of data; prevalence hypothesised; system unprepared; treat prisoners with dignity, keep safe, adequate healthcare – need for guidelines
5	Dillon et al, 2019, UK	Qualitative	To study understandings & experience of dementia in prison	Semi-structured interviews	*n* = 30 (staff = 17, prisoners = 13); 2 male sex offender prisons	n/a	Need for training, environment change, balance independence & need, social interaction, programmes, information sharing & wide support
6	du Toit & Ng, 2022, Australia	Qualitative	To examine how external organisations support PLPWD	Group discussions	*n* = 27 (legal, health and social services); 55% female, 63% > 40 years	n/a	External organisations can support training, healthcare, & information sharing, Barriers: finance, infrastructure, care-custody conflicts
7	du Toit et al, 2019, Australia	Review	To review dementia care in prison, focused on models of best practice	Scoping review	35 papers: UK (*n* = 12), Australia (*n* = 10), USA (*n* = 7), Canada (*n* = 2), France (*n* = 1), Malaysia (*n* = 1), Switzerland (*n* = 1), the UN (*n* = 1)	n/a	Care pathways useful; mixed on specialised facilities. Voluntary agencies could be used more. Barriers: finance, and facilities in community
8	Forsyth et al, 2020, UK	Mixed methods	To (i) validate a screening tool, (ii) identify gaps in service provision, and (iii) develop a care pathway for PLPWD and mild cognitive impairment	Questionnaires, semi-structured interviews, ethnography	(i) 869 prisoners (273 female); (ii) & (iii) Questionnaires: 85 governors, 77 health managers; Interviews: *n* = 42, 5 prisons (14 prisoners – 9 PLPWDs)	Validation of 6CIT screening tool; description of developing care pathways	(i)unable to validate 6CIT for prison use; (ii) low numbers screen older people at reception, or had a care pathway; (iii) all > 50 screened on reception using MoCA, further assessment if needed. Care plans shared if consented. Locate on ‘normal’ or regional specialist wings; environments to be more dementia friendly, release locations related to risk
9	Jennings, 2009, USA	Qualitative	Investigate older prisoners’ experience of health & healthcare	Semi-structured interviews	*n* = 16 (4 prisoners, 3 family, 2 clergy, 4 staff, 3 volunteers)	n/a	Possible role for social workers liaising with families of people with dementia
10	King’s Fund, 2013, UK	Mixed methods	Evaluation of the Enhancing the Healing Environment (EHE) programme	Observations, workshops, routinely collected data	10 evaluation sites (no prisons, but implemented in around 30 prisons)	The EHE programme in prisons focused on: health centre, association areas & palliative care	No outcomes for prisons specifically. Overall programmes reportedly aided decision-making, reduced agitation & distress, increased interaction & independence, safe, value for money
11	Masters et al, 2016, USA	Survey	To evaluate a staff training programme focused on ageing prisoners	Facts on Aging Quiz; study-specific questionnaire	*n* = 69 healthcare & prison staff from across the Nebraska prison system	Dementia module: symptoms, progression, behaviour, impact, challenges, management	Pre-post-quiz results not significant. Non-medical staff—new information increased understanding of behaviours; medical staff understood more about what nonmedical staff want to know
12	Moll, 2013, UK	Qualitative	To identify good practice in the management & support of prisoners with dementia	Survey	Unknown. 14 prisons’ staff or volunteers (UK = 8, USA = 4, Japan = 1, Belgium = 1)	Various – regime & accommodation adaptation; structured programmes; hospices	Some increase in wellbeing for day centre users and specialist wing residents; positive on wing exercise, forums, peer support & training. Specialist units costly, staff cuts hamper work
13	Patterson et al, 2016, Australia	Qualitative	To develop tools & procedures to assess & manage prisoners with dementia	Policy Delphi surveys; focus groups	Surveys: *n* = 36 nurses; groups: *n* = 18, (13 nurses, 2 managers, 2 OTs, 1 geriatrician)	None – the research involved developing tools & procedures	Difficulties developing one-off screening tools, need a second-stage further assessment; 2 further algorithms detailed dementia assessment, and dementia management, in prison
14	Peacock et al, 2019, Canada	Review	To review and synthesise the literature on PLPWDs’ health and social care needs	Integrative review	Eight papers: Australia (*n* = 1), France (*n* = 1), UK (*n* = 3), USA (*n* = 3)	n/a	Need assessment framework, provision varies; need to adapt environments, early advanced directives. Barriers: time, lack of training, people being drunk/high at reception
15	Soones et al, 2014, USA	Mixed methods	To assess legal professionals understanding of age-related conditions	Survey; semi-structured interviews	Survey: *n* = 71; Interviews: *n* = 10 (5 lawyers, 3 social work, 2 judges)	n/a	Knowledge gaps: health, recognising cognitive impairment, assessing safety in prison, services on release. Recommend training & checklists
16	Treacy et al, 2019, UK	Mixed methods – one-year follow-up	To evaluate a dementia friendly communities initiative in two prisons	Study-specific questionnaires; interviews; focus groups	*n* = 68 (50 prisoners, 18 staff) in two male prisons (one sex offender, one local)	Dementia Friendly Community–information sessions, meeting with dementia charity, develop action plans	Info sessions reportedly increased knowledge; one prison created an action plan with some impact on awareness, environment & independence. Nos diagnosed with dementia & use of specialist units impacted dementia friendly practice
17	Turner, 2018, USA	Qualitative	To explore prisons’ dementia assessment practices & needs	Semi-structured interviews	*n* = 7 (4 psychologists & 1 assistant;1 psychiatr-ist, 1 nurse); 3 prisons	n/a	Identified a lack of training, use of screening tools & policies for the assessment of dementia
18	Williams et al, 2012, USA	Qualitative	To identify gaps in knowledge regarding older prisoners, and develop a policy agenda	Roundtable meeting of experts	*n* = 29 (doctors, psychologists, lawyers, a nurse, prisoner advocates)	None	9 priority areas for older prisoners inc: identifying & assessing dementia (plus: definition of older & functional impairment, training, women, accommodation, release, & palliative care)
***(ii) Guidance & inspection documents***							
19	Alzheimer’s Society, 2018, UK	Guidance	To help officers understand & respond to prisoners with dementia	No methods information	n/a	None	Booklet for officers describing dementia & its impact, and tips for supporting people
20	Correctional Investigator, 2019, Canada	Inspection	To identify best prison policy & practice regarding older prisoners	Routinely collected data, interviews	*n* = 335 (280 prisoners & ex-prisoners; 55 staff & community staff)	None	Care to focus on dignity & human rights; resources should be allocated to community alternatives; need for national strategy
21	Dementia Action Allian-ce, 2017, UK	Guidance	To identify areas of need and solutions for prisoners with dementia	Roundtable discussion, but no methods	Unknown	None	Briefing note outlining challenge: diagnosis, care, routines, environment, training, & human rights
22	Department of Health, 2007, UK	Guidance	To help health & prison staff meet the needs of older prisoners	No methods information	n/a		Little dementia-focused: assessments to identify memory impairments; a dementia register should be developed, and care to be reviewed
23	Feczko, 2014, USA	Protocol	Overview of assessment, diagnosis & treatment issues for prisoners with dementia	No methods section	n/a	Clinical dementia protocol for healthcare: assessments, treatment & referral procedures	Challenge in prison to detect & treat dementia, esp with a lack of guidelines. Need collaboration across disciplines in prison, & for mainstream dementia research to include prisoners
24	Hamada, 2015, USA	Protocol	Presents an assessment and treatment protocol to be used by clinical psychologists (ATPEACE)	No methods information	n/a	Assessment & Treatment for Elders with Alzheimer’s in the Correctional Environment	Need to address lack of: dementia & risk factor awareness, diagnostic tests & evaluations, therapy & preventative strategy use, knowledge of services, cultural competence
25	Her Majesty's Inspectorate of Prisons, 2014, UK	Inspection criteria	Criteria for inspections for the treatment of women in prison	Consult staff, prisoners interest groups plus ministers	Not reported	Prison inspection with 4 tests of: safety, respect, purposeful activity & resettlement	Criteria include: healthcare staff to be dementia screening trained, & be able to recognise social care needs and dementia signs
26	Her Majesty's Inspectorate of Prisons, 2015, UK	Inspection	Inspection of HMP Isle of Wight	Observation, surveys, records	Surveys *n* = 371. Cat B prison, male, mostly sex offenders, 45.5% > 50 years	Inspected 4 tests: safety, respect, purposeful activity & resettlement	Good memory support services, regular visits by memory specialists, specialist memory-focused gym activities, & routine check-ups are booked for prisoners (such as dentist)
27	Her Majesty's Inspectorate of Prisons, 2016, UK	Inspection	Inspection of HMP Stafford	Observation, surveys, records	Surveys *n* = 196. Cat C male sex offender prison, 43.3% > 50 years	Inspected 4 tests: safety, respect, purposeful activity & resettlement	Balanced approach to disciplinary aided by clinicians & training – 20 prison staff studying for a dementia qualification (NVQ). No healthcare lead for older prisoners
28	Her Majesty's Inspectorate of Prisons, 2017a, UK	Inspection criteria	Criteria for inspections for the treatment of men in prison	No methods information	Not reported	Inspection with 4 tests: safety, respect, purposeful activity, rehabilitation & release planning	Criteria include: staff working with older prisoners to be able to recognise dementia signs; waiting times for access to memory or dementia services to be equivalent to community
29	Her Majesty's Inspectorate of Prisons, 2017b, UK	Inspection	Inspection of HMP Erlestoke	Observation, surveys, records	Surveys *n* = 149. Cat C male prison, 18.8% > 50 years	Inspected 4 tests: safety, respect, purposeful activity & resettlement	Two healthcare assistants provide an outreach service within the prison to monitor the vulnerable, including people with dementia
30	Her Majesty’s Prison Hull, 2015, UK	Prison action plan	The prisons’ plan for better outcomes for prisoners with dementia	No methods information	n/a	Improve standards via healthcare partners and staff & prisoner training	30 peer supporters trained by dementia organisation; staff demand exceeding places; staff shortages a challenge to implementation
31	Her Majesty’s Prison Littlehey, 2016, UK	Prison action plan	The prisons’ plan for better outcomes for prisoners with dementia	No methods information	n/a. Cat C prison, 2 units for men > 60 years & peer supporters	Raising awareness (staff-prisoners); environmental change; collaboration	Increased: understanding, peer support relation-ships with peers with dementia, staff- prisoner dialogue; environment: door colours, floors, seating; conference bringing groups together
32	Her Majesty’s Prison & Prob-ation Service, 2018, UK	Guidance	To present a toolkit or model of delivery, for Governors to meet needs of older prisoners	Review & case reports – no further information	n/a	Regime, environment & activity adaptation; family contact; training; peer support; palliative care;	Older prisoners a ‘specialist’ cohort. Recommend staff training; tailored regimes & inclusive activities with voluntary organisations, encourage family ties. No evidence for separate accommodations
33	Inspector of Custodial Services, 2015, Australia	Inspection	To understand current policy & practice in management & care of older prisoners	Interviews, focus groups, discussions; observations	Unknown. Interviews – managers; focus gps- staff/prisoners. 4 prisons (male + female)	n/a	Environments difficult; no needs assessments for placements; lack of structured recreational activity; staff relations positive but lack knowledge; healthcare needs not met; ad-hoc release planning
34	Ministry of Justice, 2013, UK	Guidance	To help officers manage & understand prisoners	No methods information	n/a	Guidance– case studies, signs, different diagnoses	Recommends officers refer people to healthcare if suspect dementia, or encourage self-referral
35	National Institute for Health and Care Excellence, 2017, UK	Guidance	Guidance on identifying & managing people with mental health problems in criminal justice system	Systematic reviews	n/a	Reviewed: training, assessment, intervention, and service delivery	Add cognitive question to screen; no evidence on case identification tools; no RCTs/reviews on prison rehab intervention – may need adaptation; need for staff training
36	Prisons and Probation Ombudsman, 2016, UK	Inspection	To investigate experience of prisoners with dementia, & challenges in supporting them	Fatal incident investigations – case studies	5 case studies, all male, aged 63–88	Learning lessons bulletin	Decision-making & capacity; social care responsibility; develop & share best practice; peer supporters trained & supported; risk assessments take a/c of dementia; family contact & inclusion
37	Public Health England, 2017a, UK	Guidance	Guidance for health & social care needs assess-ments for older prisoners	Consult health-justice staff & users	n/a	Guidance document	Case example of screening service; adapted cell
38	Public Health England, 2017b, UK	Guidance	Guidelines for physical health checks in prisons programmes	No methods information	n/a	Targets blood pressure, smoking, diet, alcohol, cholesterol, inactivity	Physical health check for 35–74 year olds serving > 2 years; dementia awareness raising for 65–74 year olds at this
39	Welsh Government and Ministry of Justice, 2011, UK	Guidance	To develop a care pathway for older prisoners	Consult prison-health staff, Government reps & public	Not known	Path phases: reception, 1^st^ night, assessment, re-assessment, care, transfer, release-resettlement	Dementia training for staff working with older prisoners; assessments to include dementia; access to memory clinics
40	Welsh Government, 2014, UK	Guidance	Guidance in implementing policy for mental health services for prisoners	Needs assess- ment, work- shops; consult prison service	Not known, but assessment & workshops facilitated by Public Health Wales	Implementation guidance document	Should be dementia screen, in-depth assessment, need referral routes to relevant services, staff training, importance of safeguarding
***(iii) Discussion & description papers***							
41	Ahalt et al, 2017, USA	Discussion	To reduce the use & impact of solitary confinement	No methods section	n/a	Solitary confinement	Recommends prohibition of solitary confinement for prisoners with cognitive impairments
42	Baldwin & Leete, 2012, Australia	Discussion	Discuss challenges & solutions regarding prisoners with dementia	No methods section	n/a	Specialist accommo-dation & regimes, plus community alternative	Lack of progress in Australia. Need to research prisoners, training, environment, & wider debate about how to deal with dementia
43	Booth, 2016, Canada	Discussion	To discuss the assessment & treatment of older sex offenders	No methods information	n/a	General, and also offending behaviour groups specifically	Cognitive issues may affect attendance & engagement with groups = little progress; may be need for specialised work & risk considerations
44	Brooke & Rybacka, 2020, UK	Descriptive	To describe and conduct a dementia education workshop	Design relates to workshop development only	Health care (*n* = 33), substance misuse (*n* = 5), Offender Man-agers (*n* = 7), managers (*n* = 8), officers (*n* = 15); peer support (*n* = 76)	Workshop addressed barriers and problem-solving, current initiatives, aimed to improve knowledge and empower	Barriers: bullying by prisoners, regime, environment, lack of prison – health/social care staff communication. Peer supporters trained for > 50 years support; staff & peers need empowering
45	Brown, 2014, Australia	Discussion	To report approaches to meeting needs of prisoners with dementia	No methods section	n/a no detail	Various—specialist units, training, regimes, peer support & programmes	Need legislative change to protect people with dementia; support needs to be interdisciplinary & collaborative
46	Christodoulou, 2012, UK	Discussion	To identify prison enviro-nment factors that could increase risk of dementia	No methods information	n/a	Various measures to reduce dementia risks	Recommends health promotion activities re: smoking, diet, exercise, isolation, blood pressure; screen for dementia. Challenged by poor resources
47	du Toit & Mc- Grath, 2018, Australia	Discussion	To identify areas of dementia practice & research that need focus	No methods information	n/a	The role of occupational therapy	No research; recommend work with prisoners with dementia, on occupational participation, & prisons’ environmental adaptation
48	Fazel et al, 2002, UK	Discussion	To explore the ethical implications of imprison-ing people with dementia	Discussion of case studies	Presented 2 case vignettes	Prison purpose: deter, incapacitate, symbolic, rehabilitate, retributive	Holding prisoners with dementia largely does not fit prison purposes. Questions appropriateness & legality of detaining people with dementia
49	Garavito, 2020, USA	Discussion	Overview of issues linked to underdiagnosing dementia	No methods section	n/a	n/a	Prisons offer little/no assistance, need screening, check-ups & early intervention. Barriers: communities uncomfortable with early release, nursing homes hesitant to house ex-prisoners
50	Gaston, 2018, Australia	Discussion	To highlight need for early identification of dementia and support strategies	No methods section	n/a	n/a	Screen with appropriate tool, staff training, adapt environments, dementia friendly communities, partner with external groups, release plans, develop guidelines & strategy
51	Gaston & Axford, 2018, Australia	Discussion	To raise awareness of dementia, & review identification & support strategies for prisoners	No methods information	n/a	n/a	Strategies: screen, placement in safe space, activities, peer support, develop release policy; adopt WHO healthy prison standards; staff training & collaboration; environment adaptation
52	Goulding, 2013, Australia	Discussion	To present best prison practice for older prisoners	Field visits – no further information	10 prisons (USA = 8, New Zealand = 1, Germany = 1)	Various – regime & environment adaptation, care models, hospice	Low ‘compliance’ of staff with screening; easier to adapt regimes & environments in minimal security prisons; consider segregated units & custody-care framework, issues with release
53	Hodel & Sanchez, 2013, USA	Intervention description	To describe a psychosocial programme for prisoners with dementia—SNPID	No methods information	California Men’s Colony –houses prisoners with severe cognitive impairments	Special Needs Program for Inmate-Patients with Dementia: environment & activity adaptation, peer support	Person with dementia can function in prison; quality of life increases, behavioural problems reduce; work is rewarding for programme staff; important to adjust environment or have specific units
54	Mackay, 2015, Australia	Discussion	To analyse how prisons can comply with human rights legislation	No method section	n/a	Human rights legislation	4 principles: not forcing treatment; not denying treatment & treat in an appropriate environment; equivalence; treat people with humanity & respect
55	Maschi et al, 2012, USA	Discussion	To raise awareness, discussion, research & advocacy for prisoners with dementia	No method section	n/a	Environmental adaptation, care models	Should focus on advanced care planning, care across ‘spectrum of severity’, peer support, staff training, environment change & specialist units, family role & support needs, release low risk
56	Mistry & Muhammad, 2015, USA	Discussion	Discuss whether prisons are equipped to care for people with dementia	No method section	Use of 1 case study	Environmental adaptation – specialist units, peers supporters	Should be dementia assessment, & that determines placement, peer support, staff training, dementia programmes & units, early release
57	Moore & Burtonwood, 2019, UK	Discussion	To examine whether PLPWDs’ healthcare needs are being met	No methods section	n/a	n/a	Barriers: regime, mistrust staff, healthcare access, costs, loss of social contacts, consent. Solutions: specialised units; adapt environment, early release
58	Murray, 2004, UK	Discussion	To review the conditions and care of prisoners with dementia	No method section	n/a	Regime & environment adaptation	Need for screening, risk-care balance, training, environment change, meaningful activities, ambivalence re specialised units & early release
59	Pandey et al, 2021, India	Discussion	Discussion of prison care and support	No methods section	n/a	n/a	Barriers: regime, lack of staff, time, environment, fear of repercussion, finances. Solutions: early advanced directives, assess, train, improve environment & staff co-ordination
60	Patel & Bonner, 2016, UK	Prevalence—presentation	A description of a cognitive screening service in a female prison	No methods information	55 prisoners offered screen, 18 consented, 12 completed. Closed female prison	Cognitive screen – tool used not reported All prisoners > 55 screen	Provisional dementia diagnosis in 25% screened (*n* = 3); 75% (*n* = 9) had ‘significant vascular risk factors’. Need to appropriately identify, treat, train staff – dementia friendly prisons, plan release
61	Peacock et al, 2018, Canada	Discussion	To explore the needs of, and interventions for, prisoners with dementia	No methods section	One case report	Care models, peer support, environmental change, programmes – dementia friendly prisons	Few interventions evaluated. Need knowledgeable MDTs & external groups, a long-term care model & specialist wards; screen & diagnose early, peer support, environmental adaptation; early release
62	Sfera et al, 2014, USA	Discussion	To deal with fronto-temporal dementia behavioural variant	No methods information	n/a	n/a	Recommend screening all > 55 years; use of palliative care model when placing people with dementia
63	Sindano & Swapp, 2019, UK	Intervention description—presentation	To present support available & possible for prisoners with dementia	No methods section	n/a	Awareness sessions with prisoners & staff; attend prisoner & staff forums	Increased diagnoses & national dementia helpline contact; developed an assessment referral tool, and ‘Top Tips’ booklet for officers (see paper 16)
64	Tilsed, 2019, UK	Discussion—presentation	To highlight inequalities faced by people with dementia in seldom heard groups	No methods section	n/a	Dementia Action Alliance roundtable (paper 18), ‘top tips’ booklet for officers [paper 16)	Need for systematic care pathway through the prison system, collaborative working including community groups, awareness sessions for staff & prisoners, use of ‘top tips’ as a resource
65	Vogel, 2016, USA	Discussion	An ‘argument’ for additional training in dementia for prison staff	No methods section	n/a	Crisis Intervention: signs, stages, impact, risk, manage, communication	Need for staff training in dementia, possibly as part of wider mental health training
66	Williams, 2014, UK	Intervention description	Description of a prison Cognitive Stimulation Therapy group	No methods section	n/a –aimed at people with mild-moderate dementia –male prison	Cognitive Stimulation Therapy –to maintain cognitive functioning	Reportedly enjoyed by prisoners; staff report increased socialising; difficulties with staff buy-in–increased over time. Facilitators find it rewarding
67	Wilson & Barboza, 2010, USA	Discussion	Discussion of the challenges & needs of prisoners with dementia	No methods section	n/a	None reported	Need: better early detection, to disclose (as a process), adapt environment, train staff, develop & implement non-pharmacological interventions

*HMIP* Her Majesty’s Inspectorate of Prisons, *MDT* Multi-disciplinary teams, *ADL* Activities of Daily Living, *HMIP* Her Majesty’s Inspectorate of Prisons, *NICE* National Institute for Health and Care Excellence

### Areas to consider in the support and management of PLiPWD during their time in prison and upon their release

**Fig. 2:**
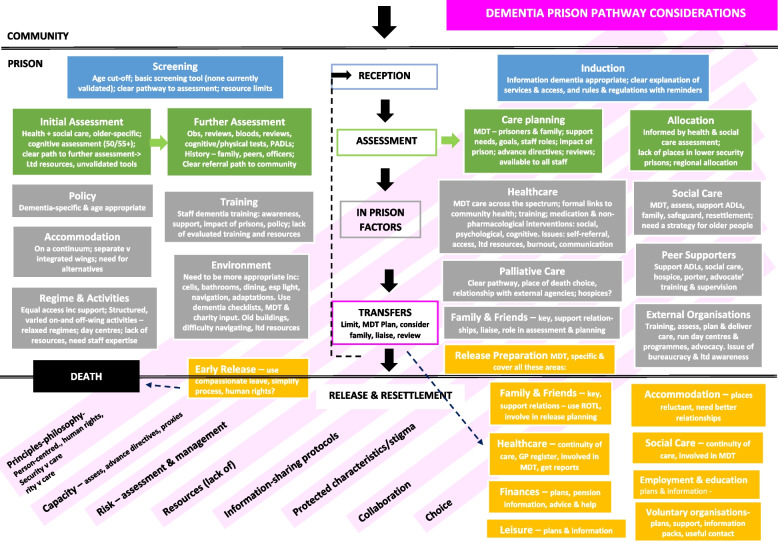
Dementia prison pathway considerations

The pathway through the prison is shown in Fig. 2, and typically involves: (i) reception into prison; (ii) assessments, and allocation of the person within prison; (iii) time held in prison; (iv) transfers between prisons, and between prisons and other services such as time spent in hospital; and (v) release and preparations for resettlement in the community. There were also a number of (vi) cross-cutting themes which could potentially impact people with dementia living in prison at each stage across the prison pathway.

#### (i) Reception

Upon entry into prison, prisoners are subject to an initial reception screening to identify and support immediate health and social care problems, and those in need of further assessment. An induction to prison rules and regulations also typically occurs at this step.

##### Screening

All papers reported that reception screening with appropriate screening tools was important in identifying cognitive difficulties and in establishing a baseline, but implementation seemed to vary (Peacock et al., 2019). One study in England and Wales found only 30% of prisons contacted routinely did this (Forsyth et al., 2020). Supporting policy and a service/person to refer to directly for further assessment were also highlighted as useful (Brooke & Jackson, 2019; Brooke et al., 2018; Gaston & Axford, 2018; Inspector of Custodial Services, 2015; Patterson et al., 2016). Proposed cut-offs for this screening were either 50 years of age (*n* = 7), under 55 years (*n* = 1), or 55 years of age (*n* = 7). One paper reported that only a third of prisoners who were offered this screening accepted it, although the reasons for this were not stated (Patel & Bonner, 2016). Another paper suggested that a screening programme could have unintended adverse consequences, that could damage already fragile relationships between staff and people living in prison (Moore & Burtonwood, 2019). Whilst many screening tools were mentioned, there are currently no tools validated for use in prisons, and many of those used in the community may be inappropriate (Baldwin & Leete, 2012; Brooke et al., 2018; du Toit et al., 2019; Feczko, 2014; Forsyth et al., 2020; Moore & Burtonwood, 2019; National Institute for Health and Care Excellence, 2017; Turner, 2018; Williams et al., 2012). One validation study found that the Six-item Cognitive Impairment Test (6CIT) was not suitably sensitive for use (Forsyth et al., 2020). Other difficulties included the limited amount of time and resources available to screen at reception (Christodoulou, 2012; Patterson et al., 2016; Peacock et al., 2019), and that staff lacked ‘familiarity’ with screening tools (Peacock et al., 2019).

##### Induction

Only two papers mentioned the induction process (Her Majesty's Prison & Probation Service, 2018; Welsh Government and Ministry of Justice, 2011) as important. A need for clearly explained information in a dementia-appropriate format (written and verbal) particularly regarding healthcare, and a recommendation that PLiPWD should be regularly reminded of rules and regulations, were suggested.

#### (ii) Assessment

Following the screening process, the current recommendation is that an initial healthcare assessment takes place in the first seven days after entering prison. During this initial assessment period, although not necessarily within this timeframe, care plans and allocation decisions may also be made regarding where the prisoner is placed within the prison.

##### Assessment

An initial older-person-specific health and/or social care assessment or standard process for assessment has been recommended by ten papers, six of which were from government or related bodies. It was also suggested by some papers, that a cognitive assessment should take place at either 50 years (*n* = 6) or 55 years (*n* = 2), which should be repeated every three months (*n* = 3), six months (*n* = 5) or annually (*n* = 12), with the latter including recommendations from NICE guidelines (National Institute for Health and Care Excellence, 2017). One study set in England and Wales found that most prisons (60%) that screened older people, did so between 7–12 months (Forsyth et al., 2020). Brief and affordable tools were considered more useful (Garavito, 2020; Turner, 2018), although the Montreal Cognitive Assessment (MOCA) was recommended in the care pathway developed by Forsyth et al. (2020).

Typically, assessments were conducted by healthcare staff, GPs or a psychologist (*n* = 6), a specialist in-house assessment unit (*n* = 2), or a specific dementia admissions assessment unit (*n* = 4). For further assessment, some prisons had internal teams to refer to (*n* = 5). Forsyth et al. (2020) recommend referral to external Memory Assessment Services for assessment. A case finding tool was being piloted in one prison (Sindano & Swapp, 2019). Assessments included can be found in Table 2.

**Table 2:** Assessments included

	**References**
Observations	(Brown, 2016; Hamada, 2015; Turner, 2018)
Clinical interviews	(Turner, 2018; Her Majesty's Prison & Probation Service, 2018; Inspector of Custodial Services, 2015; Ministry of Justice, 2013)
Record reviews	(Turner, 2018; Welsh Government and Ministry of Justice, 2011)
Physical and blood tests	(Feczko, 2014; Moll, 2013; Turner, 2018; Wilson & Barboza, 2010)
Cognitive assessment tools	(Forsyth et al. 2020; Moll, 2013; Patterson et al., 2016; Feczko, 2014; Hamada, 2015; Inspector of Custodial Services, 2015; Welsh Government and Ministry of Justice, 2011; Gaston & Axford, 2018; Goulding, 2013; Mistry & Muhammad, 2015; Sindano & Swapp, 2019; Wilson & Barboza, 2010)
Collateral histories with family	(Brooke & Jackson, 2019; Turner, 2018; Feczko, 2014; National Institute for Health and Care Excellence, 2017; Welsh Government and Ministry of Justice, 2011; Maschi et al., 2012; Wilson & Barboza, 2010)
Collateral histories with advocates	(Brooke & Jackson, 2019; National Institute for Health and Care Excellence, 2017)
Collateral histories with officers and prisoner friends	(Brooke & Jackson, 2019; Feczko, 2014; Maschi et al., 2012; Wilson & Barboza, 2010)

Assessments also explored risk and safeguarding (National Institute for Health and Care Excellence, 2017; Patterson et al., 2016; Welsh Government and Ministry of Justice, 2011), environmental impact (National Institute for Health and Care Excellence, 2017), capacity (Prison & Probation Ombudsman, 2016), work, education, and drug and alcohol use (Welsh Government and Ministry of Justice, 2011) and a person’s strengths (Hamada, 2015; National Institute for Health and Care Excellence, 2017). Prison staff contributed to some assessments of activities of daily living (ADLs) or prison-modified ADLs (Brooke et al., 2018; Brown, 2016; Dillon et al., 2019; Department of Health, 2007; Feczko, 2014; Forsyth et al., 2020; Gaston, 2018; Gaston & Axford, 2018; Patterson et al., 2016; Turner, 2018; Welsh Government and Ministry of Justice, 2011; Williams et al., 2012). Challenges to Assessment can be found in Table 3.

**Table 3:** Challenges to Assessment

There are difficulties in accessing specialists to undertake dementia assessments in the prison setting (Moore & Burtonwood, 2019). Challenges included a lack of: procedure regarding further assessment for people of concern (Brooke & Jackson, 2019; Gaston & Axford, 2018; Sindano & Swapp, 2019; Tilsed, 2019; Treacy et al., 2019; Turner, 2018); limited staff knowledge (Forsyth, Heathcote and Senior et al. 2020); staff confidence in diagnosing dementia (Sindano & Swapp, 2019; Treacy et al., 2019), including prison healthcare staff delaying diagnosis (Sindano & Swapp, 2019; Treacy et al., 2019); lack of training and, particularly in local prisons (Forsyth, Heathcote and Senior et al. 2020); regular health-checks for long-stay prisoners (Brooke & Jackson, 2019; Brown, 2016); time and resources (Turner, 2018; Correctional Investigator Canada, 2019; Inspector of Custodial Services, 2015; Goulding, 2013); the high turnover of prisoners (Forsyth, Heathcote and Senior et al. 2020) and; prison-specific screening or assessment tool(s) (Brooke & Jackson, 2019; Patterson et al., 2016; Turner, 2018; Correctional Investigator Canada, 2019; Feczko, 2014; National Institute for Health and Care Excellence, 2017). Problematically, prisoners tend to underreport any cognitive or physical symptoms either for fear of repercussions (Pandey et al., 2021) or because of poor insight into their cognitive impairment and deteriorating health (du Toit et al., 2019) and it was reported that some people did not attend assessments for fear of bullying from other prisoners (Murray, 2004). Further to this, ensuring that consent is given freely by an incarcerated individual (who may also have dementia) is challenging. Low literacy levels and high rates of learning disabilities would mean that provision of information and checking of understanding would have to be scrupulous to ensure informed consent had been obtained (Moore & Burtonwood, 2019)	

##### Care plans

Twelve papers described or recommended care planning post-assessment, in collaboration with PLiPWD and primary care, or a multi-disciplinary team (MDT) of health, social care and prison staff with external specialists healthcare proxies charities or family (Brown, 2016; Dillon et al., 2019; du Toit & Ng, 2022; Hamada, 2015; Her Majesty's Inspectorate of Prisons, 2014; Her Majesty's Prison & Probation Service, 2018; Moll, 2013; National Institute for Health and Care Excellence, 2017; Patterson et al., 2016; Prisons and Probation Ombudsman, 2016; Welsh Government and Ministry of Justice, 2011). However, it was suggested that prison staff be removed from the decision-making process as the dementia progresses, and be part of the ‘duty of care’ of healthcare staff and services (du Toit & Ng, 2022). It was recommended too that care plans be disseminated to prison wing staff (Forsyth et al., 2020) and peer supporters (Goulding, 2013), and that consent be sought for this (Goulding, 2013; Her Majesty's Inspectorate of Prisons, 2014) An ombudsman report in England and Wales noted that care plans for PLiPWD who had died in prison were inadequate (Peacock et al., 2018), and of the varying degrees of care planning found by Forsyth et al (2020), it was described typically as “rudimentary” (p26). Care plans are described further in Table 4.

**Table 4:** Care plans

• Care plans were largely described as focused on ways to support behavioural, cognitive and social difficulties, and goal setting (Brown, 2016; Hamada, 2015; National Institute for Health and Care Excellence, 2017; Prisons and Probation Ombudsman, 2016; Baldwin & Leete, 2012). However, detailing staff and peer supporter roles (Her Majesty's Prison & Probation Service, 2018) and the impact of prison (National Institute for Health and Care Excellence, 2017), whilst balancing safety with a right to quality of life (National Institute for Health and Care Excellence, 2017; Murray, 2004), and family (National Institute for Health and Care Excellence, 2017) were also recommended. Early education about advance directives and developing these was suggested (Brown, 2016; Cipriani et al. 2017; Prisons and Probation Ombudsman, 2016; Brown, 2014; Maschi et al., 2012) and an emphasis on choice (Department of Health, 2007; National Institute for Health and Care Excellence, 2017; Welsh Government and Ministry of Justice, 2011). Formulating culturally appropriate plans was also highlighted as key in one paper (Hamada, 2015). Regular reviews of the plans were also recommended (Brown, 2016; Her Majesty's Inspectorate of Prisons, 2014; Welsh Government and Ministry of Justice, 2011; Baldwin & Leete, 2012), possibly quarterly for those with high needs, or yearly for those with low needs (Brown, 2016). Care co-ordination and reviews of progress will be overseen by a dementia nurse (Forsyth, Heathcote and Senior et al. 2020). Justice health staff would track the progression of dementia-related symptoms, communicate with external health services, and refer prisoners with dementia-related cognitive impairments for discharge planning (du Toit & Ng 2022). Problematically, older prisoners’ well-being needs including the need for purpose, comfort, companionship, and quality of life are often overlooked by current prisoner dementia care efforts (du Toit & Ng 2022)	

##### Allocation

Many papers reported that prisons did or should make decisions about where people should be accommodated within the prison after health assessments (Brown, 2016; Feczko, 2014; Forsyth et al., 2020; Hodel & Sanchez, 2013; Inspector of Custodial Services, 2015; Mistry & Muhammad, 2015; Turner, 2018; Welsh Government and Ministry of Justice, 2011; Williams et al., 2012), taking age and health into account. However, despite recommendations that PLiPWD should be placed on the ground floor on low bunks for instance (Baldwin & Leete, 2012; Department of Health, 2007; Welsh Government and Ministry of Justice, 2011), there were reports that this was not happening (Inspector of Custodial Services, 2015). There were also recommendations for allocations to be made across a region to ensure people are appropriately placed in the prison system (Baldwin & Leete, 2012; Booth, 2016; Gaston & Axford, 2018; Welsh Government and Ministry of Justice, 2011). Concerns were expressed about the lack of lower category places for PLiPWD (Department of Health, 2007), and the lack of guidance regarding placement of people with high support needs (Sindano & Swapp, 2019) in England and Wales.

#### (iii) Within-prison issues

##### Policy

A number of papers reported on a need for policies or frameworks to support staff to identify, assess and support people who may be living with dementia (Brooke et al., 2018; Brooke & Jackson, 2019; Department of Health, 2007; Feczko, 2014; Gaston, 2018; Gaston & Axford, 2018; Patterson et al., 2016; Turner, 2018; Welsh Government and Ministry of Justice, 2011), without which staff have faced difficulties in providing quality care and support (Feczko, 2014; Prisons and Probation Ombudsman, 2016). Whilst there were some examples of guidance for dementia (Hamada, 2015; Patterson et al., 2016; Treacy et al., 2019; Turner, 2018), it was suggested that all policies should be reviewed and amended to ensure that they are appropriate for older people and people living with dementia (Department of Health, 2007; Lee et al., 2019; Treacy et al., 2019). Specific policy areas are described in Table 5.

**Table 5:** Policy needs

Specific policy areas needed were: a clear information sharing protocol (Dillon et al., 2019; Department of Health, 2007); an open-door policy (Brown, 2016; Cipriani et al. 2017; Treacy et al., 2019; Her Majesty's Inspectorate of Prisons, 2014; Her Majesty's Inspectorate of Prisons, 2017b; Her Majesty's Prison & Probation Service, 2018; Goulding, 2013); retirement pay commensurate with working prisoners’ rates (Treacy et al., 2019; Department of Health, 2007; National Institute for Health and Care Excellence, 2017); use of force and disciplinary procedures (Correctional Investigator Canada, 2019); resettlement strategy (Treacy et al., 2019; Department of Health, 2007); and maintaining family contact and relationships (Treacy et al., 2019; Prisons and Probation Ombudsman, 2016). The need for a comprehensive evidence base, to support policy change, was highlighted (Murray, 2004), as was the need for staff training to support implementation (Soones et al., 2014; Correctional Investigator Canada, 2019; Department of Health, 2007)	

##### Training

Issues around staff training on dementia were discussed in the majority of papers (*n* = 54) Many of these reported that prison staff either lacked training on dementia, or that training was limited (*n* = 16), with one study in England and Wales reporting that only a quarter of prison staff had received such training (Forsyth et al., 2020). Perhaps consequently, a number of papers identified that prison staff required some dementia training (*n* = 19). Staff working on a specialist dementia unit reportedly had a comprehensive 40-h training (Brown, 2014, 2016; Gaston & Axford, 2018; Hodel & Sanchez, 2013; Moll, 2013), and it was suggested that more comprehensive training be facilitated for officers, particularly those working with PLiPWD (*n* = 18) and offender managers (*n* = 2). A need for all staff working with PLiPWD to be supervised was also suggested (Gaston & Axford, 2018; Maschi et al., 2012). Despite a lack of consensus on content and duration (du Toit et al, 2019), typically, the staff training undertaken and recommended was in four areas (Table 6). It was also recommended that training for healthcare could be more comprehensive and focused on screening, identification, assessment, diagnoses, supervision and intervention training (Baldwin & Leete, 2012; Brooke & Jackson, 2019; Brown, 2014; Gaston & Axford, 2018; Her Majesty's Inspectorate of Prisons, 2014; Moll, 2013; Moore & Burtonwood, 2019; National Institute for Health and Care Excellence, 2017; Peacock et al, 2019; Treacy et al, 2019; Turner, 2018; Williams, 2014). It is of note that only 21% of healthcare staff in one study in England and Wales reported attending training to identify dementia (Forsyth et al., 2020), similar to the figures regarding prison staff in the same study.

**Table 6:** Staff training

	**References**
*Awareness and understanding*: particularly symptoms that may present as disobedience	(Brooke et al., 2018; Brooke & Jackson, 2019; Brooke & Rybacka, 2020; Brown, 2016; Cipriani et al. 2017; Dillon et al., 2019; du Toit & Ng 2022; Forsyth, Heathcote and Senior et al. 2020; Masters et al., 2016; Moll, 2013; Moore & Burtonwood, 2019; Pandey et al., 2021; Peacock et al., 2019; Soones et al., 2014; Treacy et al., 2019; Turner, 2018; Williams et al., 2012; Alzheimer's Society, 2018; Correctional Investigator Canada, 2019; Dementia Action Alliance, 2017; Department of Health, 2007; Feczko, 2014; Her Majesty's Inspectorate of Prisons, 2014; Her Majesty's Inspectorate of Prisons, 2016; Her Majesty's Inspectorate of Prisons, 2017b; HMP Hull, 2015; HMP Littlehey, 2016; Her Majesty's Prison & Probation Service, 2018; Inspector of Custodial Services, 2015; Ministry of Justice, 2013; National Institute for Health and Care Excellence, 2017; Prisons and Probation Ombudsman, 2016; Welsh Government and Ministry of Justice, 2011; Brown, 2014; Gaston, 2018; Gaston & Axford, 2018; Goulding, 2013; Hodel & Sanchez, 2013; Maschi et al., 2012; Mistry & Muhammad, 2015; Sindano & Swapp, 2019; Tilsed, 2019; Vogel, 2016)
*Support*: *minimising confusion and agitation, and communication skills*	(Brooke & Jackson, 2019; Brown, 2016; Cipriani et al. 2017; du Toit & Ng 2022; Masters et al., 2016; Moll, 2013; Turner, 2018; Alzheimer's Society, 2018; Correctional Investigator Canada, 2019; Dementia Action Alliance, 2017; Department of Health, 2007; HMP Littlehey, 2016; Her Majesty's Prison & Probation Service, 2018; Inspector of Custodial Services, 2015; National Institute for Health and Care Excellence, 2017; Brown, 2014; du Toit & McGrath, 2018; Gaston, 2018; Gaston & Axford, 2018; Hodel & Sanchez, 2013; Maschi et al., 2012; Mistry & Muhammad, 2015; Peacock et al., 2018; Vogel, 2016; Wilson & Barboza, 2010)
*The impact of the prison environment and culture including regime, stigma and exploitation*	(Pandey et al., 2021; Treacy et al., 2019; Turner, 2018; Alzheimer's Society, 2018; Her Majesty's Prison & Probation Service, 2018; National Institute for Health and Care Excellence, 2017; Welsh Government and Ministry of Justice, 2011; Vogel, 2016)
*Training to support policy, principles and legislation*	(Treacy et al., 2019; Williams et al., 2012; National Institute for Health and Care Excellence, 2017; Welsh Government and Ministry of Justice, 2011; Gaston, 2018; Mackay, 2015; Maschi et al., 2012; Mistry & Muhammad, 2015; Vogel, 2016; Williams, 2014; Wilson & Barboza, 2010)

Much of the training described in the included papers had been formulated and delivered by dementia- or older people-specific voluntary organisations (Alzheimer’s Society, 2018; Brooke et al. 2018; Brown, 2016; Gaston & Axford, 2018; HMP Hull, 2015; Her Majesty's Prison & Probation Service, 2018; Hodel & Sanchez, 2013; Moll, 2013; Peacock et al., 2018; Prisons and Probation Ombudsman, 2016; Sindano & Swapp, 2019; Tilsed, 2019; Treacy et al., 2019). Although it has also been recommended to involve health and social care (Goulding, 2013; Her Majesty's Prison & Probation Service, 2018; Ministry of Justice, 2013; Treacy et al., 2019; Turner, 2018), and officers and peer supporters (Brooke & Jackson, 2019; Masters et al., 2016; National Institute for Health and Care Excellence, 2017; Treacy et al., 2019) in developing the training. In one study, prison staff were also trained to deliver dementia information sessions to their peers (Treacy et al., 2019). A suggestion of video-training packages was also made (du Toit et al., 2019). Dementia training typically lacked robust evaluation (Brooke et al., 2018), although those available generally reported benefits in their understanding of dementia, relationships, and diagnoses (Goulding, 2013; HMP Littlehey, 2016; Masters et al., 2016; Sindano & Swapp, 2019; Treacy et al., 2019). It was also reported that some prison staff were resistant to working with PLiPWD (Moll, 2013), and that resource limitations resulted in training cuts (HMP Hull, 2015; Treacy et al., 2019).

##### Healthcare

Offering healthcare across the spectrum for PLiPWDs, from acute to chronic care, with a focus on preventative and long-term care as well as palliative care was recommended by some papers (Brown, 2014; du Toit & Ng, 2022; Gaston, 2018; Maschi et al., 2012; Mistry & Muhammad, 2015; Peacock et al, 2018; Welsh Government and Ministry of Justice, 2011; Williams et al., 2012). The development of care pathways to guide this were also recommended or formulated (du Toit et al., 2019; Forsyth et al., 2020; Peacock et al., 2019), although the majority (69%) of prisons in one study in England and Wales did not have one (Forsyth et al., 2020). Clear and formal links with local hospitals, memory clinics, forensic and community teams for planning, training, advice, support and in-reach were also present or recommended by sixteen research and guidance papers. The amount of healthcare cover in prisons in England and Wales reportedly varied with the function of the prison with largely only local prisons having 24-h healthcare staff (Treacy et al., 2019), and most other forms of prison having office-type hours’ healthcare cover – including sex offender prisons where the majority of older prisoners are held (Brown, 2016; Correctional Investigator Canada, 2019; Goulding, 2013; Inspector of Custodial Services, 2015; Treacy et al., 2019). While specialist services or units for PLiPWD exist in a number of jurisdictions (Baldwin & Leete, 2012; Brown, 2016; Cipriani et al., 2017; Gaston & Axford, 2018; Goulding, 2013; Hodel & Sanchez, 2013; Inspector of Custodial Services, 2015; Maschi et al., 2012; Mistry & Muhammad, 2015; Treacy et al, 2019), more are reportedly needed (Brooke et al., 2018; du Toit et al., 2019; Forsyth et al., 2020; Welsh Government and Ministry of Justice, 2011).

Most healthcare teams were reportedly MDT, or this was recommended, alongside joint health and social care working (*n* = 16). A number of healthcare staff acted as the lead for older people in prisons (Department of Health, 2007; Her Majesty's Inspectorate of Prisons, 2014; Her Majesty's Inspectorate of Prisons, 2016; Moll, 2013; Welsh Government and Ministry of Justice, 2011), with a recommendation that a dementia-trained nurse should lead any dementia care pathways (Forsyth et al., 2020) and indeed it was suggested that healthcare staff in general have training and experience in working with older people (Her Majesty's Inspectorate of Prisons, 2014; Her Majesty's Inspectorate of Prisons, 2017b; Moll, 2013; Patterson et al., 2016; Public Health England, 2017b; Treacy et al., 2019; Turner, 2018; Welsh Government and Ministry of Justice, 2011). Whilst one of the recommended roles for healthcare was the prescription and monitoring of medication (Feczko, 2014; Her Majesty's Inspectorate of Prisons, 2017b; Moll, 2013), much of the focus was on early identification and diagnosis, and keeping a dementia register (Department of Health, 2007; Moll, 2013; Patterson et al., 2016; Welsh Government and Ministry of Justice, 2011), and the use of non-pharmacological approaches. These broadly included: psychological interventions (Goulding, 2013; Hamada, 2015; Moll, 2013; Wilson & Barboza, 2010); assistance with ADLs and social care (Feczko, 2014; Hamada, 2015; Hodel & Sanchez, 2013; Maschi, et al., 2012; Murray, 2004; Prisons and Probation Ombudsman, 2016); development and delivery of specialist dementia prison programmes (Brown, 2014, 2016; Hodel & Sanchez, 2013; Mistry & Muhammad, 2015; Moll, 2013; Peacock et al., 2018; Wilson & Barboza, 2010); reablement and rehabilitation (Welsh Government and Ministry of Justice, 2011); relaxation (Wilson & Barboza, 2010); safeguarding (Hodel & Sanchez, 2013); and cognitive stimulation groups (Moll, 2013; Williams, 2014). Other possible roles included: training or supporting staff and peer supporters, as reported in fourteen papers, as well as advocacy (Feczko, 2014; Peacock et al., 2018; Welsh Government and Ministry of Justice, 2011), allocation, assessment for offending behaviour groups, risk assessments and disciplinary hearings (Booth, 2016; Department of Health, 2007; Her Majesty's Prison & Probation Service, 2018; Murray, 2004; Prisons and Probation Ombudsman, 2016). Challenges to Healthcare are noted in Table 7.

**Table 7:** Challenges to Healthcare

Challenges included: conflicting priorities of custodial and care frameworks (du Toit & Ng 2022) a lack of intervention evaluations or reviews to inform practice (Treacy et al., 2019; National Institute for Health and Care Excellence, 2017; Gaston & Axford, 2018); a lack of resources (specialists, escort staff and money) (Inspector of Custodial Services, 2015; Welsh Government and Ministry of Justice, 2011; Fazel et al., 2002); staff resistance (Turner, 2018); lack of understanding of the prison context (Gaston & Axford, 2018; Williams, 2014); high levels of staff burnout (Gaston & Axford, 2018); people not accessing healthcare for fear of bullying (Cipriani et al. 2017); not being able to physically access healthcare centres (Treacy et al., 2019; Her Majesty's Inspectorate of Prisons, 2017b; Welsh Government and Ministry of Justice, 2011, Gaston & Axford, 2018); limited access to healthcare services (Moore & Burtonwood, 2019); delays in arranging assessments (Forsyth, Heathcote and Senior et al. 2020); diagnosis and/or the provision of care (Forsyth, Heathcote and Senior et al. 2020) and; healthcare staff lacking access to prisoners at night (Welsh Government and Ministry of Justice, 2011). There are barriers for nurses to develop therapeutic relationships with those they care for due to correctional requirements and the physical environment, affecting nurse–patient relationship building (Pandey et al., 2021). There may also be a mistrust of prison healthcare staff (Moore & Burtonwood, 2019). The mental health services are often focused on other inmates whose behaviours are more challenging (Pandey et al., 2021). There were multiple issues around referrals, with some people not ‘able’ to self-refer (Prisons and Probation Ombudsman, 2016), prison staff can be a barrier, and so referrals should not have to go through them (Treacy et al., 2019; Her Majesty's Prison & Probation Service, 2018), although it was also suggested that healthcare staff should accept referrals from prison staff as they are the prison frontline (Brown, 2016; Moll, 2013; Treacy et al., 2019; Ministry of Justice, 2013; Prison and Probation Ombudsman 2016, Welsh Government and Ministry of Justice 2011]. Cognitive decline is also a barrier to providing health care in prison from the help-seeking side is a further impediment (Pandey et al., 2021). One suggestion was that healthcare staff automatically book in check-up appointments (Her Majesty's Inspectorate of Prisons, 2015), and one prison ran an in-reach programme of healthcare assistants worked on prison wings to identify concerns (Her Majesty's Inspectorate of Prisons, 2017a)	

##### Palliative care

A care pathway for dying people that meets community standards was recommended (Department of Health, 2007; Her Majesty's Prison & Probation Service, 2018; Welsh Government and Ministry of Justice, 2011), as was ensuring that people could choose a preferred place to die (Her Majesty's Prison & Probation Service, 2018). Some prisoners were moved to community hospices or hospitals (Brooke & Jackson, 2019; Inspector of Custodial Services, 2015), or it was felt that they should be (Her Majesty's Prison & Probation Service, 2018). Although it was noted that some prisons lack relationships with community hospices or palliative care services and need to foster them (Brooke & Jackson, 2019; Brown, 2016; Correctional Investigator Canada, 2019; Department of Health, 2007; Her Majesty's Prison & Probation Service, 2018).

A number of prisons also reportedly had hospices, particularly in the United States (Brooke et al., 2018; Brown, 2016; Feczko, 2014; Goulding, 2013; Williams et al., 2012), although these have not been comprehensively evaluated (Williams et al., 2012). It was recommended that these be staffed by MDTs (Her Majesty's Prison & Probation Service, 2018), including chaplains and nutritionists (Her Majesty's Prison & Probation Service, 2018; Goulding, 2013), and many included prisoner peer supporters (Brooke et al., 2018; Goulding, 2013). The use of independent contractors was also suggested as staff-prisoner relationships were considered problematic in some prisons (Williams et al., 2012). Regarding family, many hospices were described as allowing more visits (Brooke & Jackson, 2019; Goulding, 2013; Her Majesty's Prison & Probation Service, 2018), including one prison with family accommodation (Her Majesty's Prison & Probation Service, 2018). Whilst re-engaging with family was reportedly encouraged (Brown, 2016), a lack of support was noted (Correctional Investigator Canada, 2019). Suggested improvements include a family liaison officer, providing a list of counselling options, and hosting memorial services (Her Majesty's Prison & Probation Service, 2018).

##### Social care

A social care strategy for older prisoners and a social care lead for all prisons in England and Wales has been recommended (Department of Health, 2007; Prisons and Probation Ombudsman, 2016). It was reported that MDTs working with PLiPWD should and increasingly do include social workers including specialist units and hospices (Baldwin & Leete, 2012; Brooke et al., 2018; Brown, 2016; Cipriani et al., 2017; Goulding, 2013; HMP Littlehey, 2016; Her Majesty's Prison & Probation Service, 2018; Maschi et al., 2012; Prisons and Probation Ombudsman, 2016; Sindano & Swapp, 2019; Treacy et al., 2019; Welsh Government and Ministry of Justice, 2011). Social care roles can be found in Table 8.

**Table 8:** Social care roles

These roles included: social care assessments (Treacy et al., 2019); family liaison and support (Jennings, 2009); supporting people with their ADLs (Department of Health, 2007; Welsh Government and Ministry of Justice, 2011; Hodel & Sanchez, 2013; Maschi et al., 2012); incontinence care (Forsyth et al. 2020); input to disciplinary proceedings and safeguarding (Her Majesty's Prison & Probation Service, 2018; Welsh Government and Ministry of Justice, 2011; Hodel & Sanchez, 2013); support, advice and training for prison staff (Her Majesty's Prison & Probation Service, 2018) and; release and resettlement (Soones et al., 2014; Department of Health, 2007; Her Majesty's Prison & Probation Service, 2018)	

The work may be direct or may be through co-ordinating external agencies or peer supporters (Brooke & Jackson, 2019; Department of Health, 2007; Her Majesty's Prison & Probation Service, 2018; Prisons and Probation Ombudsman, 2016; Tilsed, 2019; Treacy et al., 2019; Welsh Government and Ministry of Justice, 2011). Clarity in these roles was considered paramount, particularly as uncertainty reportedly continues to exist over who is responsible for meeting prisoners’ social care needs in some prisons in England and Wales despite the passing of the Care Act, 2014 (Dementia Action Alliance, 2017; Tilsed, 2019; Welsh Government and Ministry of Justice, 2011). There was also some ambiguity around the threshold PLiPWD were expected to meet in order to access social care (Forsyth et al., 2020). In some instances, personal care was delivered informally by untrained and unsupported prison staff and peer supporters in lieu of suitably trained social care workers (Treacy et al., 2019), with issues raised about the unavailability of social care through the night (Forsyth et al., 2020). Where social care staff were involved in coordinating personal care for prisoners, it was reported as positive for prisoners and prison staff (Her Majesty's Inspectorate of Prisons, 2016; Treacy et al., 2019), particularly, in one prison, where social care staff were prison-based (Forsyth et al., 2020).

##### Peer supporters

Prisoner peer supporters were operating in a number of prisons, as reported in 22 papers, and their employment was recommended by a further fourteen. Typically, these were people who had ‘good’ disciplinary and mental health records, and certainly in the US, were longer-serving prisoners. A number of papers indicated the need for peer supporters to receive training in dementia, including awareness and support (Brooke et al., 2018; Brooke & Jackson, 2019; Brown, 2016; Correctional Investigator Canada, 2019; Department of Health, 2007; Dillon et al., 2019; du Toit & Ng, 2022; Gaston, 2018; Gaston & Axford, 2018; Goulding, 2013; HMP Hull, 2015; HMP Littlehey, 2016; Her Majesty's Prison & Probation Service, 2018; Inspector of Custodial Services, 2015; Maschi et al., 2012; Mistry & Muhammad, 2015; Sindano & Swapp, 2019; Tilsed, 2019; Treacy et al., 2019). Comprehensive 36–40 h training on dementia was delivered for those working on specialist units, including one leading to a qualification (Brooke & Jackson, 2019; Brown, 2016; Gaston & Axford, 2018; Her Majesty's Prison & Probation Service, 2018; Moll, 2013). Much of the training was developed and delivered by charities, particularly dementia-related ones, as reported in eleven papers. Ongoing support and supervision was offered or recommended by some prisons, provided largely by health or social care staff or charities (Brooke & Jackson, 2019; Brown, 2016; Correctional Investigator Canada, 2019; Department of Health, 2007; Her Majesty's Prison & Probation Service, 2018; Gaston & Axford, 2018; Maschi et al., 2012; Prisons and Probation Ombudsman, 2016; Sindano & Swapp, 2019; Treacy et al., 2019), with informal peer-to-peer support also described (Brown, 2016; Gaston & Axford, 2018; Treacy et al., 2019). The support and supervision received was found to be valuable (Brooke & Jackson, 2019; Brown, 2016; Treacy et al., 2019). Peer-supporter roles are listed in Table 9.

**Table 9:** Peer-supporter roles

The roles that peer supporters played regarding PLiPWD included: social/personal care and support with ADLs (Brooke & Jackson, 2019; Brooke & Rybacka, 2020; Brown, 2016; Forsyth, Heathcote and Senior et al. 2020; Moll, 2013; Pandey et al., 2021; Treacy et al., 2019; HMP Littlehey, 2016; Her Majesty's Prison & Probation Service, 2018; Inspector of Custodial Services, 2015; Prisons and Probation Ombudsman, 2016; Welsh Government and Ministry of Justice, 2011; Brown, 2014; Gaston & Axford, 2018; Goulding, 2013; Hodel & Sanchez, 2013; Maschi et al., 2012; Mistry & Muhammad, 2015; Peacock et al., 2018); ‘portering’ (Moll, 2013; Treacy et al., 2019; Her Majesty's Prison & Probation Service, 2018; Prisons and Probation Ombudsman, 2016; Goulding, 2013; Mistry & Muhammad, 2015); supporting prison wellbeing and support programmes (du Toit & McGrath, 2018; Goulding, 2013; Hodel & Sanchez, 2013; Mistry & Muhammad, 2015); gym work and a social environment (Brooke & Rybacka, 2020); hospice work (Brooke et al., 2018; Brown, 2016; Moll, 2013; Goulding, 2013); facilitators (Pandey et al., 2021) and; advocacy (Goulding, 2013; Treacy et al., 2019). In one paper peer supporters were considered a part of the prison MDT (du Toit & McGrath, 2018)	

A number of benefits to: (a) the peer supporters, (b) the prisoners they supported and, (c) the prison, were described, although formal evaluations were lacking (Brown, 2016; Christodoulou, 2012; Department of Health, 2007; du Toit et al., 2019; Gaston, 2018; Gaston & Axford, 2018; Goulding, 2013; Treacy et al., 2019; Welsh Government and Ministry of Justice, 2011). This included: payment, development of skills which could be used on release, positive impact on progression through the system, and on self-confidence and compassion, and the creation of a more humane environment. However, frustration and distress amongst peer supporters largely when untrained and unsupported was also reported (Brooke & Jackson, 2019; Brown, 2016; Correctional Investigator Canada, 2019; Inspector of Custodial Services, 2015; Prisons and Probation Ombudsman, 2016; Treacy et al., 2019), and concerns raised in relation to an over-reliance on peers to do work that it is the statutory duty of health and social care to provide (Prisons and Probation Ombudsman, 2016; Treacy et al., 2019). This was a particular problem in light of personal care being prohibited for peer supporters in England and Wales (Her Majesty's Prison & Probation Service, 2018; Moll, 2013). It is also of note that the role of peer supporter may also attract the opprobrium of other prisoners, with reports that they have been seen as ‘snitches’ or ‘dogs’ in some areas (Brown, 2016; Goulding, 2013). In addition, in some prisons, the peer supporter role was not advocated due to: fear of litigation; fear of replacing staff with peers; belief that people should be acquiring more transferable skills, since many would be unable to undertake care work in the community due to their offence history (Brown, 2016; Goulding, 2013).

##### Accommodation

There were mixed views regarding accommodation for PLiPWD. A continuum of prison accommodation was suggested from independent to 24-h care (including assisted living) (Forsyth et al., 2020; Gaston & Axford, 2018; Williams et al., 2012). A number of papers (*n* = 18) recommended that there should be some form of alternative, more appropriate accommodation developed, potentially regional, including secure facilities possibly with a palliative orientation (Hodel & Sanchez, 2013; Mistry & Muhammad, 2015; Sfera et al., 2014). However, there were concerns about the availability, costs and staffing of specialist units, and distances that family would have to travel to visit despite potential benefits (du Toit et al., 2019; Moore & Burtonwood, 2019). It was also suggested that PLiPWD should be released to live in the community instead (Correctional Investigator Canada, 2019).

Within prisons, there was a debate evident within the papers about whether PLiPWD should be accommodated in separate units or integrated within the general prison population, which had generated little clear evidence and mixed views (Brooke & Jackson, 2019; Dillon et al., 2019; Her Majesty's Prison & Probation Service, 2018; Treacy et al., 2019). Authors have suggested that specialist or separate wings focused on older people or those with dementia were safer, met peoples’ needs better, and offered better care, support and programmes than integrated units (Brown, 2014; Dillon et al., 2019; du Toit & Ng, 2022; du Toit et al., 2019; Goulding, 2013; Maschi et al., 2012; Murray, 2004; Treacy et al., 2019; Williams et al., 2012), as long as they were ‘opt-in’ for prisoners and staff (Correctional Investigator Canada, 2019; Moll, 2013; Treacy et al., 2019; Williams et al., 2012), and opportunities to get off the wing to socialise with others are provided (Treacy et al., 2019). The types of ‘specialist’ accommodation that PLiPWD were living in are reported in Table 10. It is of note that papers reported a highly limited number of beds available in specialist units (Inspector of Custodial Services, 2015; Patterson et al., 2016; Turner, 2018), and that a number of older prisoner-specific prisons were being closed due to costs (Turner, 2018).

**Table 10:** Types of ‘specialist’ accommodation that prisoners with dementia currently reside

Residence	References
Prisons specifically for older prisoners only (= 4) mostly in the United States	(Baldwin & Leete, 2012; Brown, 2014; Goulding, 2013; Jennings, 2009)
Separate wings or blocks for older prisoners without specific care staff or facilities (*n* = 5)	(Treacy et al., 2019; HMP Littlehey, 2016; Welsh Government and Ministry of Justice, 2011; Gaston & Axford, 2018; Murray, 2004)
Separate wings or blocks for older prisoners with care staff and facilities (*n* = 4)	(Brown, 2016; Welsh Government and Ministry of Justice, 2011; Gaston & Axford, 2018; Goulding, 2013)
Prisons care-type facilities for prisoners with disabilities or care needs (*n* = 8)	(Brown, 2016; Treacy et al., 2019; Correctional Investigator Canada, 2019; Inspector of Custodial Services, 2015; Baldwin & Leete, 2012; Brown, 2014; Gaston & Axford, 2018; Goulding, 2013)
Specific units for PLiPWD or cognitive difficulties in three prisons (*n* = 7), all in the United States	(Brown, 2016; Correctional Investigator Canada, 2019; Brown, 2014; Gaston & Axford, 2018; Goulding, 2013; Hodel & Sanchez, 2013; Maschi et al., 2012)

Four papers described the benefits of older people and those PLiPWD residing within the general prison population (Dillon et al., 2019; Her Majesty's Prison & Probation Service, 2018; Treacy et al., 2019; Williams et al., 2012). Those living with dementia reported a benefit from socialising with, and being cared for by, younger people (Dillon et al., 2019; Her Majesty's Prison & Probation Service, 2018; Williams et al., 2012). The presence of older people also reportedly calmed younger prisoners (Dillon et al., 2019; Her Majesty's Prison & Probation Service, 2018; Williams et al., 2012). Importantly, removing people from their prison social networks may have a detrimental effect (Williams et al., 2012), and living on specialist units can be stigmatising (Treacy et al., 2019).

##### Regime and activities

The maintenance of prisons regimes is the primary focus of prison officers (Brooke & Jackson, 2019). However, there was a reported need (*n* = 19) for PLiPWD to have equal access to activities and services including work, education, gym, library and day centres where they exist, as well as a structured and varied regime on the wing on which they were accommodated, and support to access these. This support could include providing adequate seating (Welsh Government and Ministry of Justice, 2011), or giving prisoners more time to accomplish activities, and to assist if needed (Brooke & Jackson, 2019; Goulding, 2013; Her Majesty's Prison & Probation Service, 2018; Hodel & Sanchez, 2013). Other recommendations included an overall relaxation of regimes (Gaston & Axford, 2018; Treacy et al., 2019), an ‘open door’ policy (Brown, 2016; Cipriani et al., 2017; Goulding, 2013; Her Majesty's Inspectorate of Prisons, 2014; Her Majesty's Inspectorate of Prisons, 2017b; Her Majesty's Prison & Probation Service, 2018; Treacy et al., 2019), more visible staff (The King's Fund, 2013), and creating a more communal social environment (Christodoulou, 2012). On-wing social activities are described in Table 11.

**Table 11:** On-wing social activities

On-wing social activities PLPWD are or reportedly should be facilitated including: bingo, crafts, chess, cards, games, gym, music, poetry, books, art, memorabilia, walking (including off-wing socialising), table tennis, Wii and air hockey (Brown, 2016; Dillon et al., 2019; The King's Fund, 2013; Forsyth, Heathcote and Senior et al. 2020; Treacy et al., 2019; Department of Health, 2007; Her Majesty's Inspectorate of Prisons, 2014; Her Majesty's Inspectorate of Prisons, 2017b; Her Majesty's Prison & Probation Service, 2018; Welsh Government and Ministry of Justice, 2011; Baldwin & Leete, 2012; Brown, 2014; Christodoulou, 2012; Goulding, 2013; Maschi et al., 2012; Mistry & Muhammad, 2015; Murray, 2004). Physical stimulation and exercise were also considered important (Brown, 2016; Moll, 2013; Her Majesty's Prison & Probation Service, 2018; Brown, 2014; Christodoulou, 2012; du Toit & McGrath, 2018; Gaston, 2018; Maschi et al., 2012), with special, adapted and separate gym activities recommended (Brooke & Jackson, 2019; Treacy et al., 2019; Department of Health, 2007; Her Majesty's Prison & Probation Service, 2018; Welsh Government and Ministry of Justice, 2011; Gaston, 2018; Gaston & Axford, 2018; Goulding, 2013), including yoga, pilates and tai chi (Moll, 2013; Department of Health, 2007), badminton and bowls (Moll, 2013), chair-based exercises (Moll, 2013), and activities to assist memory (Her Majesty's Inspectorate of Prisons, 2015). Rehabilitation activities (Goulding, 2013), therapeutic activities (Brown, 2016; Goulding, 2013; Maschi et al., 2012], reminiscence or life stories (Dillon et al., 2019; Moll, 2013; Brown, 2014; du Toit & McGrath, 2018; Goulding, 2013) memory cafes, holistic care and support, an over ‘45 s’ focus group, over 50 s well-being and mindfulness (Forsyth, Heathcote and Senior et al. 2020), sensory stimulation (Hodel & Sanchez, 2013), and cognitive stimulation groups (Forsyth, Heathcote and Senior et al. 2020; Treacy et al., 2019; Her Majesty's Prison & Probation Service, 2018; Sindano & Swapp, 2019) have also been provided and recommended. It was also noted that it would be useful for people in prison LWD to have some autonomy such as being able to prepare drinks and snacks for themselves (Dementia Action Alliance, 2017; Hodel & Sanchez, 2013; Maschi et al., 2012; Treacy et al., 2019)	

Having on-wing work available or alternative means for prisoners who are unable to work to make money was also reportedly important (Christodoulou, 2012; Department of Health, 2007; Gaston, 2018; Gaston and Axford, 2018; Her Majesty's Inspectorate of Prisons, 2014, 2016, 2017b; Her Majesty's Prison & Probation Service, 2018; Moll, 2013; Murray, 2004; Treacy et al., 2019; Welsh Government and Ministry of Justice, 2011). It was suggested that people with dementia should have the chance to work if wanted, and adaptations could be made to work programmes or working days made shorter to facilitate this. Some prisons had specific roles which involved lighter, simple, repetitive tasks such as gardening (Baldwin & Leete, 2012; Brooke & Jackson, 2019; Inspector of Custodial Services, 2015; Moll, 2013; Treacy et al., 2019). Day centres existed in some prisons, or were thought to be feasible (Department of Health, 2007; Her Majesty's Prison & Probation Service, 2018; Moll, 2013; Treacy et al., 2019; Welsh Government and Ministry of Justice, 2011), and it was suggested that attendance at these could constitute meaningful paid activity (Her Majesty's Prison & Probation Service, 2018). The centres were largely developed and facilitated by charities, and ran a wide variety of social, therapeutic, recreational, arts and advice-centred activities (Her Majesty's Prison & Probation Service, 2018; Moll, 2013).

Equal access to educational activities, including rehabilitation and offending behaviour programmes, was highlighted as important, particularly where attendance is needed to facilitate people’s progression through the system (Booth, 2016; Brooke & Jackson, 2019; Dillon et al., 2019; Department of Health, 2007; Her Majesty's Prison & Probation Service, 2018). Some prisons provided, or felt there was a need for, particular educational activities for PLiPWD and adaptations may be, or have been, made to learning materials and equipment, content and pace (Brooke & Jackson, 2019; Department of Health, 2007; Gaston, 2018; Gaston & Axford, 2018; Her Majesty's Prison & Probation Service, 2018; Treacy et al., 2019; Welsh Government and Ministry of Justice, 2011). Dedicated library sessions have been designated in some prisons, and some libraries can and do stock specialist resources including books, audiobooks, reminiscence packs and archives of local photos, music and DVDs (Department of Health, 2007; Her Majesty's Prison & Probation Service, 2018;Treacy et al., 2019; Williams, 2014). Educational materials could and have been available between sessions to aid memory with distance learning also possible (Brooke & Jackson, 2019; Her Majesty's Prison & Probation Service, 2018). Suggestions for alternatives for PLiPWD focused on activity and stimulation (du Toit & Ng, 2022; Gaston, 2018; Her Majesty's Prison & Probation Service, 2018), preparing for retirement classes (Department of Health, 2007), health promotion (Brooke et al., 2018; Christodoulou, 2012; Gaston & Axford, 2018; Her Majesty's Prison & Probation Service, 2018; Maschiet al., 2012; Murray, 2004; Welsh Government and Ministry of Justice, 2011), the arts (Brooke & Jackson, 2019) and IT classes (Her Majesty's Prison & Probation Service, 2018). Prisoner forums or representative could also be consulted regarding regimes and activities (Moll, 2013; Her Majesty's Prison & Probation Service, 2018; Welsh Government and Ministry of Justice, 2011). Challenges to regimen and activities are described in Table 12.

**Table 12:** Challenges to regimen and activities

Some of the challenges to delivering an equal but adapted regime and activities include a lack of resources, especially staff time (Brooke & Jackson, 2019; Inspector of Custodial Services, 2015; Treacy et al., 2019; Turner, 2018), the need for (and lack of) dedicated key workers (Feczko, 2014; Welsh Government and Ministry of Justice, 2011), dementia leads or champions across the prison (Sindano & Swapp, 2019), and a designated activities co-ordinator (Ministry of Justice, 2013; Mistry & Muhammad, 2015). Staff and prisoners identified the prison regime, with extended periods of time behind locked doors as a challenge (Brooke & Rybacka, 2020). Restrictions to regimes and activities due to security conditions were also noted (Brown, 2016; Goulding, 2013; Inspector of Custodial Services, 2015), as well as some activities being physically inaccessible (Dementia Action Alliance, 2017; Inspector of Custodial Services, 2015). Staff resistance to some activities was also reported (Williams, 2014). A number of papers reported that there was a lack of prison activities and programmes overall (Brown, 2016; Treacy et al., 2019; Her Majesty's Prison & Probation Service, 2018; Inspector of Custodial Services, 2015; Baldwin & Leete, 2012; Christodoulou, 2012; Goulding, 2013; Mistry & Muhammad, 2015; Murray, 2004; Peacock et al., 2018)	

##### Environment

A large number (*n* = 42) of the included papers discussed changes that prisons had made, or should make, to the built environment in order to be more suitable for PLiPWD – in one study in England and Wales, around half of prisons surveyed had made such environmental modifications (Forsyth et al., 2020). These focused on: (i) prisoners’ cells, (ii) bathrooms, (iii) dining hall, (iv) outside space and recreation areas, and (v) overall general prison environment (Table 13).

**Table 13:** Environment

Setting	Adaptations	References
**Prisoners’ cells**	Dementia-adapted	Prisons and Probation Ombudsman, 2016; Public Health England, 2017a
	Single	Brown, 2016; Treacy et al., 2019; Inspector of Custodial Services, 2015
	Accessible	Forsyth et al. 2020; Treacy et al., 2019; Public Health England, 2017a; Williams et al., 2012; Her Majesty's Prison & Probation Service, 2018; Maschi et al., 2012; Wilson & Barboza, 2010
	On the ground floor	Treacy et al., 2019; Prisons and Probation Ombudsman, 2016; Public Health England, 2017a; Goulding, 2013; Department of Health, 2007; Gaston & Axford, 2018
	Identifiable (use of colour, pictures, name tags)	Brown, 2016; Hodel & Sanchez, 2013
	No mirrors	Alzheimer's Society, 2018, Her Majesty's Prison & Probation Service, 2018; Brown, 2014; Sindano & Swapp, 2019; Wilson & Barboza, 2010
	Adjustable low beds	Brown, 2016; Treacy et al., 2019; Goulding, 2013; Williams et al., 2012; Department of Health, 2007; Inspector of Custodial Services, 2015; Gaston & Axford, 2018; Mistry & Muhammad, 2015
	Extra bedding and clothing	Department of Health, 2007; Her Majesty's Prison & Probation Service, 2018; Wilson & Barboza, 2010
	Using Velcro on clothing (du Toit et al., 2019)	
	Issuing slip-on shoes (du Toit et al., 2019)	
	A bathroom	Brown, 2016
	An in-cell alarm system	Forsyth, Heathcote and Senior et al. 2020; Correctional Investigator Canada, 2019; Department of Health, 2007; Her Majesty's Prison & Probation Service, 2018; Welsh Government and Ministry of Justice, 2011
**Bathrooms**	Adapted, easy-to-access bathrooms	Brown, 2016; Treacy et al., 2019; Correctional Investigator Canada, 2019; Department of Health, 2007; Feczko, 2014; Gaston & Axford, 2018; Goulding, 2013; Maschi et al., 2012
	Arrows to low toilets with different coloured seating	Brown, 2016; The King's Fund, 2013; Williams et al., 2012
	Signs for handwashing	Brown, 2016
	Use of commodes	du Toit et al., 2019
	Handrails in showers	du Toit et al., 2019
**Dining hall**	Communal and homely dining halls that are easy to access	Brown, 2016; Treacy et al., 2019; The King's Fund, 2013; Hodel & Sanchez, 2013; Williams et al., 2012; Department of Health, 2007
**Outside space and recreation areas**	Accessible outside space	Brown, 2016; Treacy et al., 2019; The King's Fund, 2013; Goulding, 2013
	Accessible recreation or social spaces	Brown, 2016; Treacy et al., 2019; The King's Fund, 2013; Goulding, 2013; Department of Health, 2007; Inspector of Custodial Services, 2015; Welsh Government and Ministry of Justice, 2011; Gaston, 2018
**Overall general prison environment**	(Natural) light	Brown, 2016; Forsyth, Heathcote and Senior et al. 2020; The King's Fund, 2013; Goulding, 2013; Cipriani et al. 2017; Moll, 2013; Alzheimer's Society, 2018; Feczko, 2014; Public Health England, 2017a; Maschi et al., 2012; Sindano & Swapp, 2019; Wilson & Barboza, 2010
	Ease of navigation and clear signage	Brown, 2016; Forsyth, Heathcote and Senior et al. 2020; Treacy et al., 2019; The King's Fund, 2013; Prisons and Probation Ombudsman, 2016; Public Health England, 2017a; Her Majesty's Prison & Probation Service, 2018; Dillon et al., 2019; Moll, 2013; Alzheimer's Society, 2018; Feczko, 2014; HMP Littlehey, 2016; Brown, 2014; Gaston, 2018; Gaston & Axford, 2018; Goulding, 2013; Maschi et al., 2012; Murray, 2004; Sindano & Swapp, 2019; Wilson & Barboza, 2010
	Hand/grab rails and assistive devices	du Toit et al., 2019; Forsyth, Heathcote and Senior et al. 2020; Treacy et al., 2019; Williams et al., 2012; Forsyth et al. 2020; Moll, 2013; Correctional Investigator Canada, 2019; Department of Health, 2007; Inspector of Custodial Services, 2015; Welsh Government and Ministry of Justice, 2011; Brown, 2014; Gaston, 2018; Gaston & Axford, 2018; Goulding, 2013; Maschi et al., 2012; Wilson & Barboza, 2010
	Longhandled equipment	Forsyth, Heathcote and Senior et al. 2020
	Level, matte, non-slip flooring	Brown, 2016; Treacy et al., 2019; The King's Fund, 2013; HMP Littlehey, 2016; Brown, 2014; Gaston, 2018; Goulding, 2013
	Magnifying screens	Forsyth, Heathcote and Senior et al. 2020
	White walls with colours identifying areas	du Toit et al., 2019; Treacy et al., 2019; The King's Fund, 2013; Her Majesty's Prison & Probation Service, 2018; Cipriani et al. 2017; Dillon et al., 2019; Moll, 2013; HMP Littlehey, 2016; Prisons and Probation Ombudsman, 2016; Brown, 2014; Goulding, 2013; Maschi et al., 2012; Wilson & Barboza, 2010
	Wide corridors	du Toit et al., 2019; Inspector of Custodial Services, 2015; Public Health England, 2017a; Gaston & Axford, 2018; Goulding, 2013
	Ramps, wheelchair accessibility and stair lifts	Forsyth, Heathcote and Senior et al. 2020; Treacy et al., 2019; Her Majesty's Prison & Probation Service, 2018; Dementia Action Alliance, 2017; Department of Health, 2007; Prisons and Probation Ombudsman, 2016; Welsh Government and Ministry of Justice, 2011; Gaston & Axford, 2018; Wilson & Barboza, 2010
	Resting points and comfortable seating	Treacy et al., 2019; The King's Fund, 2013; Dementia Action Alliance, 2017; Department of Health, 2007; HMP Littlehey, 2016; Inspector of Custodial Services, 2015; Gaston, 2018
	Large wing clocks and calendars	Brown, 2016; The King's Fund, 2013; Prisons and Probation Ombudsman, 2016; Public Health England, 2017a; Dillon et al., 2019; Brown, 2014; Hodel & Sanchez, 2013
	Seasonal or nature-oriented art and age-appropriate memorabilia	The King's Fund, 2013
	Noise reduction measures	Her Majesty's Prison & Probation Service, 2018
	Temperature control	Goulding, 2013

Problematically, the age and dementia-inappropriateness of buildings are considered a challenge (Baldwin & Leete, 2012; Brown, 2016; Dementia Action Alliance, 2017; Forsyth et al., 2020; Goulding, 2013; Inspector of Custodial Services, 2015; Mistry & Muhammad, 2015; Prisons and Probation Ombudsman, 2016; Treacy et al., 2019). Difficulties in navigating prisons where everywhere looks the same (Dementia Action Alliance, 2017; Murray, 2004; Treacy et al., 2019), and the lack of budget (HMP Littlehey, 2016; HMP Littlehey, 2016; Inspector of Custodial Services, 2015; Treacy et al., 2019) were also reported issues. It was suggested that the use of dementia-friendly environmental checklists could be useful, potentially with input from occupational therapists, health and social care, and dementia charities and in-house education, work and estates departments (Brown, 2014; Christodoulou, 2012; Dillon et al., 2019; Goulding, 2013; HMP Littlehey, 2016; Her Majesty's Prison & Probation Service, 2018; Hodel & Sanchez, 2013; Peacock et al., 2018; Sindano & Swapp, 2019; Treacy et al., 2019). Hope was expressed that newly built prisons would be more dementia-friendly (Dementia Action Alliance, 2017; Her Majesty's Prison & Probation Service, 2018; Williams et al., 2012).

##### Family

Formal policies and procedures should be in place to help maintain links between family and prisoners, and to foster an understanding of the central importance of families particularly for PLiPWD (Her Majesty's Inspectorate of Prisons, 2016; Treacy et al., 2019). Some papers described how prisons could support contact by: giving help and additional time to make telephone calls and arranging visits in quieter spaces (Her Majesty's Prison & Probation Service, 2018; Prisons and Probation Ombudsman, 2016; Treacy et al., 2019); increasing the number of visits (Jennings, 2009); and allowing for accumulated visits or transfers to other prisons for visits closer to home (Her Majesty's Prison & Probation Service, 2018). Family communication – additional information can be found in Table 14.

**Table 14:** Family communication

A number of papers also described finding ways for families and prisons to communicate by initially seeking permission from prisoners to talk to their families (Brown, 2016; du Toit & Ng 2022; Welsh Government and Ministry of Justice, 2011), by involving family in assessments, planning and support (National Institute for Health and Care Excellence, 2017; Prisons and Probation Ombudsman, 2016; Maschi et al., 2012) and helping with the disclosure of diagnoses to prisoners (Feczko, 2014; National Institute for Health and Care Excellence, 2017; Maschi et al., 2012; Wilson & Barboza, 2010). The use of a charity or social worker as a liaison between families and the prisons was proposed, as a means of reporting concerns (Dillon et al., 2019; Jennings, 2009; Patterson et al., 2016; Treacy et al., 2019; Her Majesty's Prison & Probation Service, 2018), and of providing support to families (Gaston, 2018; Maschi et al., 2012; Peacock et al., 2018; Treacy et al., 2019). However, some prisons did not support prisoners to maintain family contact, when it would be relatively simple to do so (Treacy et al., 2019; Correctional Investigator Canada, 2019; Prisons and Probation Ombudsman, 2016; Mistry & Muhammad, 2015). One paper suggested that prisons may lack awareness of support available for families (Hamada, 2015), and another that privacy regulations may preclude family involvement (Feczko, 2014). It was also recommended that distance from family be considered when transferring prisoners (Her Majesty's Prison & Probation Service, 2018)	

##### External organisations

One review suggested that external voluntary agencies were not often contacted or referred to, despite their potential benefits in terms of costs and support for staff and PLiPWDs (du Toit et al., 2019). However, other papers reported that charities for PLiPWD, or older people, were involved in (or were recommended to be involved in): designing and/or delivering dementia training; being part of MDTs; informing the design of referral processes, screening, assessment and case finding tools; consulting on environmental design; creating and delivering social care plans (including running activity centres); advice and support; advocacy and; co-facilitating a cognitive stimulation therapy group (Alzheimer’s Society 2018; Brooke et al., 2018; Brown, 2014, 2016; Correctional Investigator Canada, 2019; Department of Health, 2007; du Toit & Ng, 2022; du Toit et al., 2019; Gaston, 2018; Gaston & Axford, 2018; Goulding, 2013; Her Majesty's Inspectorate of Prisons, 2014; HMP Hull, 2015; Her Majesty's Prison & Probation Service, 2018; Hodel & Sanchez, 2013; Moll, 2013; Peacock et al., 2018; Prisons and Probation Ombudsman, 2016; Sindano & Swapp, 2019; Tilsed, 2019; Treacy et al., 2019; Williams, 2014). It was also recommended that external organisations need to have a better knowledge and understanding of prisons and people living in prison, in order to better manage risk, and for clear information sharing protocols (du Toit & Ng, 2022).

#### (iv) Transfers

During the course of their sentence, people in prison may be transferred to other prisons for various reasons or to receive treatment in hospital. The need for MDT transfer plans to be developed was reported (Welsh Government and Ministry of Justice, 2011), as was the need to limit the number of prisoner transfers as moving accommodation is likely to have an adverse effect (Her Majesty's Prison & Probation Service, 2018; Patterson et al., 2016). It was recommended that transfers should take the distance from family and friends into account (Her Majesty's Prison & Probation Service, 2018), and that the ‘receiving’ facility (prison or healthcare setting) should be liaised with regarding health and social care, and risk (Her Majesty's Prison & Probation Service, 2018; Welsh Government and Ministry of Justice, 2011) to ensure continuity of care (Cipriani et al., 2017). A standard document transfer protocol was also postulated as useful, as documents need to be forwarded quickly as well (Brown, 2016; Tilsed, 2019; Welsh Government and Ministry of Justice, 2011). At the receiving facility, it was suggested that assessments and care plans should be reviewed on the day of the transfer (Brown, 2016; Her Majesty's Prison & Probation Service, 2018; National Institute for Health and Care Excellence, 2017; Welsh Government, 2014), and for re-inductions to be facilitated for prison transfers (Her Majesty's Prison & Probation Service, 2018).

#### (v) Release and resettlement

Most prisoners will be released from prison at the end of their sentence, although a number may die before their time is served. A number of areas were highlighted regarding the release and resettlement of PLiPWD, including the possibility of early release due to dementia.

##### Early release

A number of papers advocated for compassionate release policies and their actual use, or alternative custodial placements such as halfway houses or secure nursing homes, that would effectively result in the early release of PLiPWD (Brown, 2016; Cipriani et al., 2017; Correctional Investigator Canada, 2019; Dementia Action Alliance, 2017; Department of Health, 2007; du Toit & Ng, 2022; du Toit et al., 2019; Fazel et al., 2002; Gaston & Axford, 2018; Goulding, 2013; Her Majesty's Prison & Probation Service, 2018; Hodel & Sanchez, 2013; Inspector of Custodial Services, 2015; Maschi et al., 2012; Mistry & Muhammad, 2015; Pandey et al., 2021; Turner, 2018; Williams et al., 2012). Although, it has also been noted that early release may not be a popular idea for some sections of the community (du Toit et al., 2019; Garavito, 2020), it was also suggested that raising community awareness of dementia may ameliorate this (du Toit & Ng, 2022). It was reported that prisoners with dementia should be considered in any criteria set forth for early release, particularly given the high cost/low risk ratio which they represent (Baldwin & Leete, 2012; Correctional Investigator Canada, 2019; Department of Health, 2007; Goulding, 2013; Her Majesty's Prison & Probation Service, 2018; Inspector of Custodial Services, 2015; Maschi et al., 2012; Murray, 2004; Williams et al., 2012). For prisoners who do not understand the aims of prison, continuing to hold them may be a contravention of human rights and equality laws – particularly where health and social care is inadequate (Baldwin & Leete, 2012; Dementia Action Alliance, 2017; Fazel et al., 2002; Gaston & Axford, 2018; Murray, 2004). It was also emphasised that the existence of units and programmes for PLiPWD should not be used to legitimise prison as an appropriate place for PLiPWD (Correctional Investigator Canada, 2019). More information can be found in Table 15.

**Table 15:** Early release

The complexity, bureaucracy and length of the early release process typically results in prisoners not being approved for release or dying before they do so (Baldwin & Leete, 2012; Brown, 2016; Inspector of Custodial Services, 2015; Maschi et al., 2012; Patterson et al., 2016; Peacock et al., 2018; Turner, 2018; Williams et al., 2012), with the process ‘over-focusing’ on risk despite increasing frailties (Goulding, 2013; Peacock et al., 2018; Williams et al., 2012), concern about malingering (Murray, 2004), and of foregrounding victims’ rights (Baldwin & Leete, 2012). Finding suitable alternative accommodation and establishing risk assessment protocols (Moore & Burtonwood, 2019) can be problematic. While in the USA, staff can refer to POPS (Feczko, 2014; Mackay, 2015), there is no equivalent in England and Wales. It has been suggested that human rights organisations could raise awareness of PLPWD in prison, and the complexity of the process that could enable their early release (Maschi et al., 2012)	

##### Resettlement

Ten different areas were identified in the literature which related to the issues PLiPWD leaving prison may face on their release and resettlement into the community, these were:

- (a) In-prison release preparation

Specific pre-release programmes or services for older people or those living with dementia may be required (Department of Health, 2007; Williams et al., 2012), with prisoners being cognitively screened prior to release (Goulding, 2013), although the latter was only found in 10% of prisons in one study (Forsyth et al., 2020). Other suggestions for programme content included: self-efficacy, health, staving off dementia and associated anxiety, accessing services, addressing institutionalisation, setting up email addresses, and the provision of information packs on national, regional and local services and resources (Department of Health, 2007; Her Majesty's Prison & Probation Service, 2018; Williams et al., 2012).

It has been suggested that release plans and transitions be facilitated by an MDT including prisoners, the voluntary sector, offender managers, and other appropriate community-based organisations (du Toit et al., 2019; Feczko, 2014; Goulding, 2013; Her Majesty's Prison & Probation Service, 2018; Inspector of Custodial Services, 2015; Moll, 2013; Welsh Government and Ministry of Justice, 2011). Recommended plan content included: risk management strategies, health, social care, housing, finance, employment, leisure and voluntary sector considerations (Welsh Government and Ministry of Justice, 2011). It was also suggested that Circles of Support and Accountability (CoSA), primarily associated with sex offenders, could be set up for PLiPWD as a means to support those leaving prison and settling back into the community particularly without family support (Her Majesty's Prison & Probation Service, 2018).

Challenges to release preparation were identified as: a lack of resources, (Turner, 2018) the lack of clarity regarding staff resettlement roles (Inspector of Custodial Services, 2015), and the lack of resettlement provision offered at sex offender prisons in England and Wales (Her Majesty's Prison & Probation Service, 2018).

- (b) Family

A number of papers reported the key role that family and friends can or do play in supporting PLiPWD leaving prison, and that this should be supported or facilitated by prison staff (Brown, 2016; Her Majesty's Prison & Probation Service, 2018; Goulding, 2013). Initially this could include encouraging diagnosis disclosure (Dillon et al., 2019), using prison leave to maintain relationships (Her Majesty's Prison & Probation Service, 2018), involvement in discharge planning (Welsh Government and Ministry of Justice, 2011), and placing prison leavers close to family upon release and ensuring family are supported (Correctional Investigator Canada, 2019; Gaston & Axford, 2018). Where PLiPWD lack family, setting up CoSAs as described above may be useful (Her Majesty's Prison & Probation Service, 2018).

- (c) Probation

It was suggested that probation staff should have training to work with older people, and that some offender managers could specialise in this work (Department of Health, 2007; Welsh Government and Ministry of Justice, 2011). Probation officers or offender managers are or can be involved in resettlement planning, (Her Majesty's Prison & Probation Service, 2018; Welsh Government and Ministry of Justice, 2011), arranging accommodation (Inspector of Custodial Services, 2015), liaising with agencies such as health care or social services, checking that PLiPWD are accessing these services and disseminating reports of to-be released prisoners to relevant parties (Department of Health, 2007; Moll, 2013; Welsh Government and Ministry of Justice, 2011). Importantly, the forwarding of important documents to offender managers by the prison should be routine (Department of Health, 2007; Moll, 2013). It was also recommended that probation staff should visit people in prison before release if they live out of area (Department of Health, 2007). The work of probation services was reportedly hampered by limited resources (Brown, 2016).

- (d) Health

Continuity of care upon release can be difficult, and it was suggested that it could be a role of prison healthcare to ensure this (including registering with the local GP and dentist (Cipriani et al., 2017; Department of Health, 2007; Gaston, 2018; Gaston & Axford, 2018; Her Majesty's Prison & Probation Service, 2018; Inspector of Custodial Services, 2015; Welsh Government and Ministry of Justice, 2011). There appeared to be some differences regarding the distribution of full healthcare reports to offender managers and other appropriate agencies with some prisons sending them, some only if requested, and some not providing them on grounds of confidentiality (Moll, 2013). Typically, it was recommended that it was better for to-be released older prisoners if these reports were disseminated (Department of Health, 2007). It was also suggested that healthcare staff in prison and from the community form part of multi-disciplinary release planning, and that these plans include health considerations and healthcare staff advice on issues of accommodation (du Toit & Ng, 2022; Inspector of Custodial Services, 2015; Moll, 2013; Welsh Government and Ministry of Justice, 2011).

- (e) Social care

Some papers reported that social workers can and should be involved in the process of resettlement (Department of Health, 2007; Welsh Government and Ministry of Justice, 2011) and release preparation (Goulding, 2013). Continuity of social care arranged with the local authority was also recommended (Her Majesty's Prison & Probation Service, 2018; Welsh Government and Ministry of Justice, 2011).

- (f) Accommodation

Release planning should include plans for accommodation, and involve housing agencies or care services in the community in that planning (Welsh Government and Ministry of Justice, 2011). Importantly, people in prison may need help in registering for housing, and their homes may be in need of adaptation in response to their health or social care needs (Department of Health, 2007; Her Majesty's Prison & Probation Service, 2018). Nursing homes and other care providing facilities were reported to be reluctant to accommodate people who have been in prison (Brown, 2014; Brown, 2016; Booth, 2016; Correctional Investigator Canada, 2019; du Toit et al., 2019; Gaston, 2018; Garavito, 2020; Goulding, 2013; Inspector of Custodial Services, 2015). This was described as particularly the case for those who were living with dementia (Brown, 2014; Correctional Investigator Canada, 2019; Dillon et al., 2019), with further issues reported in accommodating those who have committed sex offences (Brown, 2014, 2016; Dillon et al., 2019; Garavito, 2020; Inspector of Custodial Services, 2015). Concerns regarding the safety of other residents and the views of their families, and the rights of victims in general, were cited as reasons behind these placement difficulties (Brown, 2014; Goulding, 2013) – one paper reported that there had been community protests (Brown, 2016).

It was suggested that prisons need to build better relationships with care providers in the community, which had reportedly been forged by some (Brown, 2016; Goulding, 2013; Inspector of Custodial Services, 2015), and that they could also provide education and support to these services (Booth, 2016). However, it was also noted that there may be a need for specialist residential units to be created in the community for people released from prison with dementia (Inspector of Custodial Services, 2015), with an example of a state-run facility for ex-prisoners in the United States (Goulding, 2013), and particular attention for younger ex-prisoners with dementia (Brown, 2014). A number of papers reported that if accommodation could not be arranged for people, this largely resulted in them remaining in prison until it was (Correctional Investigator Canada, 2019; Goulding, 2013; Inspector of Custodial Services, 2015; Peacock et al., 2018; Soones et al., 2014).

- (g) Finance

Imprisonment likely leads to a loss of income, meaning that older prisoners who may have served more lengthy sentences are likely to be poorer, particularly if unable to work in prison (Baldwin & Leete, 2012; Gaston, 2018). Therefore, it was suggested that release planning ought to include issues of finance (Welsh Government and Ministry of Justice, 2011). Given that it has been suggested that people in prison should be given advice on pensions and welfare benefits, and help to arrange these (Department of Health, 2007; Her Majesty's Prison & Probation Service, 2018; Goulding, 2013), addressing this would seem to be an area of particular use for older people leaving prison who may have additional problems in these areas, and for those who may need assistance in arranging their financial affairs because of their deteriorating health problems.

- (h) Employment and education

People’s employment prospects are likely to be impacted upon release from prison, particularly for older people who may have served long sentences (Gaston, 2018). Where appropriate, it was recommended that release planning should include issues around employment (Welsh Government and Ministry of Justice, 2011), that information packs for people should include sections on education and employment, and that it could be useful to help people make links with the Department for Work and Pensions (Her Majesty's Prison & Probation Service, 2018).

- (i) Leisure

Leisure activities and resources could be considered in release planning, and included in pre-release information packs for prisoners (Her Majesty's Prison & Probation Service, 2018; Welsh Government and Ministry of Justice, 2011).

- (j) Charities and voluntary sector organisations

It was recommended in a number of papers that charity and voluntary sector organisations working with PLiPWD be involved in release planning (Department of Health, 2007; du Toit et al., 2019; Her Majesty's Prison & Probation Service, 2018; Moll, 2013; Welsh Government and Ministry of Justice, 2011), continuity of care (Moll, 2013), and in providing support during the transition and after (du Toit & Ng, 2022; Welsh Government and Ministry of Justice, 2011). It was also suggested that in general it would be useful for PLiPWD to have contact with these organisations (Department of Health, 2007; Her Majesty's Prison & Probation Service, 2018; Inspector of Custodial Services, 2015), and that they may be well-placed to develop information packs for prisoners on release regarding local amenities, services and resources (Her Majesty's Prison & Probation Service, 2018).

#### (vi) Cross-cutting themes

Eight more generalised concerns were also described which had a clear impact on the passage of PLiPWD through prison, on release and resettlement in the community, and on the issues raised thus far in the review.

##### Principles-philosophy

The principles suggested to underpin the support of PLiPWD are that it should be person-centred, holistic, adhere to human rights and dignity principles, proactive, health promoting, and enabling – making choices but supported if needed (Brown, 2014, 2016; Christodoulou, 2012; Cipriani et al., 2017; Correctional Investigator Canada, 2019; Department of Health, 2007; Dillon et al., 2019; du Toit & Ng, 2022; Gaston & Axford, 2018; Her Majesty's Inspectorate of Prisons, 2017b; Her Majesty's Prison & Probation Service, 2018; Mackay, 2015; Maschi et al., 2012; Treacy et al., 2019; Welsh Government and Ministry of Justice, 2011; Wilson & Barboza, 2010). Conversely, clashes in philosophies between prison staff, and health and social care staff have been reported with security trumping care in many cases, which can have a negative impact (du Toit & Ng, 2022; Gaston, 2018; Gaston & Axford, 2018; Goulding, 2013; Mackay, 2015; Murray, 2004; Patterson et al., 2016; Prisons and Probation Ombudsman, 2016; Treacy et al., 2019; Williams, 2014). It was suggested that positioning dementia as more than just a health issue and fostering a whole-prison care-custody model or approach, with clearly defined roles for ‘care’ and ‘custody’, may be useful in resolving this (du Toit & Ng, 2022; Public Health England, 2017b; Welsh Government and Ministry of Justice, 2011).

##### Resources

A number of papers (*n* = 15) reported that budget and resource limitations had a variety of negative impacts including difficulties in providing: appropriate assessment, support and accommodation to PLiPWD; specialist accommodations, plans for which were then curtailed; delivering programmes and activities; healthcare cover; and, staff training (Booth, 2016; Christodoulou, 2012; Correctional Investigator Canada, 2019; Dementia Action Alliance, 2017; Dillon et al., 2019; du Toit et al., 2019; du Toit & Ng, 2022; Goulding, 2013; HMP Hull, 2015; Jennings, 2009; Mackay, 2015; Moll, 2013; Moore & Burtonwood, 2019; Pandey et al., 2021; Patterson et al., 2016; Peacock et al., 2018; Treacy et al., 2019; Turner, 2018). Ultimately, lack of resources has reportedly led to a system that is not able to cope appropriately with PLiPWD (Moll, 2013; Williams et al., 2012; Wilson & Barboza, 2010), with associated problems transferring out of the prison system into probation and care systems when people are released (Williams et al., 2012).

##### Capacity

It has been suggested that PLiPWD in prison should be treated as if they have capacity to make decisions such as giving or withholding consent for treatment, unless it is proven otherwise. This is consistent with legislation such as the Mental Capacity Act (Prisons and Probation Ombudsman, 2016). It has been recommended that healthcare staff should conduct capacity assessments if there are concerns (National Institute for Health and Care Excellence, 2017; Welsh Government and Ministry of Justice, 2011), and be trained to do so (Maschi et al., 2012; Welsh Government, 2014). It is of note that an ombudsman report showed that PLiPWD who died lacked access to mental capacity assessments (Peacock et al., 2018). For PLiPWD, who are likely to lack capacity as their condition progresses, early education about, and development of, advance directives has been advocated (Brown, 2016; Cipriani et al., 2017; Inspector of Custodial Services, 2015; Maschi et al., 2012; Prisons and Probation Ombudsman, 2016), and staff should be trained on this (Maschi et al., 2012). It has also been suggested that family members, independent mental capacity advocates or healthcare proxies could or should be used for PLiPWD who lack capacity in making care, welfare and financial decisions (Brown, 2016; Soones et al., 2014), supported by legislation and oversight, as opposed to prison or healthcare staff making decisions (Correctional Investigator Canada, 2019).

##### ‘Risk’

The issue of ‘risk’ related to PLiPWD revolves around four areas: (i) assessment, (ii) management, (iii) disciplinary procedures, and (iv) safeguarding. Full details can be found in Table 16.

**Table 16:** Risk

Area	Issues	Recommendations
**Assessment**	Prison classification systems do not make allowances for the mostly lowered risk of older people, and those LWD (Inspector of Custodial Services, 2015)	Risk assessments should be comprehensive and individualised to consider age and the impact of health on future offending (National Institute for Health and Care Excellence, 2017; Prisons and Probation Ombudsman, 2016; Welsh Government and Ministry of Justice, 2011; Booth, 2016; du Toit & McGrath, 2018; Goulding, 2013)
	There are conflicting recommendations about the use of assessment tools (National Institute for Health and Care Excellence, 2017; Booth, 2016)	Risk assessments should be undertaken by skilled staff (Brooke et al., 2018; Hamada, 2015; National Institute for Health and Care Excellence, 2017; Booth, 2016)
		Risk assessments should be reviewed regularly (National Institute for Health and Care Excellence, 2017; Prisons and Probation Ombudsman, 2016)
**Management**		The need to balance safety with the need for risk-taking (Brown, 2016; Murray, 2004)
		The need for training regarding PLPWD (Brown, 2016; Murray, 2004)
**Disciplinary process**		Prison policies and procedures regarding disciplinary procedures and the use of restraint and force be modified for older people, the frail, and those LWD (Treacy et al., 2019; Correctional Investigator Canada, 2019; Her Majesty's Prison & Probation Service, 2018; Prisons and Probation Ombudsman, 2016; Welsh Government and Ministry of Justice, 2011; Peacock et al., 2018)
		Staff training (Treacy et al., 2019; Correctional Investigator Canada, 2019; Her Majesty's Inspectorate of Prisons, 2016)
		A balanced approach to disciplinary procedures, with the need to discern between dementia and ‘bad’ behaviour (Dillon et al., 2019; Alzheimer's Society, 2018; Her Majesty's Inspectorate of Prisons, 2016; Her Majesty's Prison & Probation Service, 2018)
		The prohibition of the use of solitary confinement or segregation for PLPWD (Ahalt et al., 2017)
**Safeguarding**	Lack of supervision for PLPWD in prison may leave them at risk (Mackay, 2015)	The need for safeguarding arrangements for older people in prison and those LWD (National Institute for Health and Care Excellence, 2017; Welsh Government and Ministry of Justice, 2011; Welsh Government, 2014; Hodel & Sanchez, 2013; Mackay, 2015)
	Bullying of prisoners with dementia by other prisoners (Brooke & Rybacka, 2020)	Training (Williams, 2014)
		A means for people to report abuse from both prisoners and staff was suggested (Welsh Government, 2014)
		Contacts for legal professionals with safeguarding concerns and training in the area (Soones et al., 2014)

There were a number of additional facets to risk concerns regarding PLiPWD described in the papers. There were concerns that the lack of understanding of the impact of dementia on people’s behaviour could ultimately lead to people being held in prison for longer periods on account of seemingly transgressive or aggressive behaviour that could in fact be related to their dementia difficulties (Dementia Action Alliance, 2017; Mistry & Muhammad, 2015; Treacy et al., 2019). In one study, a prisoner with dementia was transferred to another prison because staff felt that they were ‘grooming’ an officer (Treacy et al., 2019), likely lengthening their overall prison stay. There was also a recurring issue in fatal incidents investigations in England and Wales of prisoners being restrained whilst dying in hospital, a practice described as unnecessary in light of their likely frail state (Peacock et al., 2018; Prisons and Probation Ombudsman, 2016). One paper suggested linking future accommodation options and considerations for Release on Temporary Licence to a PLiPWD’s risk of reoffending, as well as the severity of their symptoms (Forsyth et al., 2020). Moore and Burtonwood (2019) also observed that a lack of risk assessment protocols was a barrier to release of PLiPWD., and as Table 16 suggests, a comprehensive risk assessment, applied by appropriately trained staff should make health and its impact on future offending more salient to aid this.

##### Choice

There were recommendations that PLiPWD should have the opportunity to make choices in their treatment and care. This included input into care plans or making informed decisions about their care (Department of Health, 2007; du Toit & Ng, 2022; National Institute for Health and Care Excellence, 2017; Welsh Government and Ministry of Justice, 2011), as well as developing advance directives particularly early in a person’s sentence (Brown, 2016; Cipriani et al., 2017; Inspector of Custodial Services, 2015; Maschi et al., 2012; Pandey et al., 2021; Peacock et al., 2019; Prisons and Probation Ombudsman, 2016), and choosing ‘preferred’ places to die (Her Majesty's Prison & Probation Service, 2018).

##### Protected characteristics

There was a reported need for culturally appropriate assessments, treatment and activities (Brooke et al., 2018; Department of Health, 2007; Hamada, 2015; Welsh Government and Ministry of Justice, 2011), spiritual support (Welsh Government and Ministry of Justice, 2011), multilingual information (Welsh Government and Ministry of Justice, 2011), and the recognition of gender differences in dementia healthcare needs (Brown, 2014; Department of Health, 2007; Williams et al., 2012). It was also highlighted that racism makes the experience of living with dementia in prison more problematic (Brooke et al., 2018; Brown, 2014; Correctional Investigator Canada, 2019). There were some examples of policy and practice within prisons which considered some protected characteristics: assessment tools in different languages (Patterson et al., 2016), additional support for PLiPWD to plan care (Department of Health, 2007; Welsh Government and Ministry of Justice, 2011), and the development of culturally appropriate care planning (Hamada, 2015). Hamada (2015) also advocated assessment and treatment that was culturally ‘competent’ and respectful, and which acknowledged the importance of culture and diversity.

An overall need to tackle dementia- and age-related stigma was also reported in some papers, and the need to foster cultures that are age-respectful should be reflected in staff training (Department of Health, 2007; Treacy et al., 2019; Welsh Government and Ministry of Justice, 2011), In addition, practices which openly discriminate such as the lack of: dedicated dementia resources (Turner, 2018), appropriate lower category prison places (Department of Health, 2007; Welsh Government and Ministry of Justice, 2011), and appropriate accommodation on release, which at times prevents release, should also be challenged (Correctional Investigator Canada, 2019; Forsyth et al., 2020; Ministry of Justice, 2013; Prisons and Probation Ombudsman, 2016). There was also a lack of research into the interaction between protected characteristics and dementia in prison (Brooke & Jackson, 2019; Treacy et al., 2019; Williams et al., 2012).

##### Collaboration

Many papers advocated the need for prisons and specialist dementia units to adopt a collaborative MDT approach drawing from staff teams across the prison regarding: the identification and support of prisoners with dementia, care planning, the disciplinary process, the development, dissemination and implementation of policy, and in environmental change and the building of new prisons (Brooke et al., 2018; Brown, 2014, 2016; Christodoulou, 2012; Cipriani et al., 2017; Dillon et al., 2019; Department of Health, 2007; Feczko, 2014; Forsyth et al., 2020; Gaston & Axford, 2018; Her Majesty's Inspectorate of Prisons, 2014, 2016; HMP Hull, 2015; HMP Littlehey, 2016; Her Majesty's Prison & Probation Service, 2018; Inspector of Custodial Services, 2015; Moll, 2013; Patterson et al., 2016; Peacock et al., 2018; Peacock, 2019; Prisons and Probation Ombudsman, 2016; Sindano & Swapp, 2019; The King’s Fund 2013; Tilsed, 2019; Treacy et al., 2019; Welsh Government and Ministry of Justice, 2011, 2014; Williams, 2014). There were examples of prisoners collaborating with staff in the care of PLiPWD as peer supporters, and having joint staff-prisoner supervision and training (Brooke & Jackson, 2019), of joint staff-prisoner wing meetings in one prison (Treacy et al., 2019), and of the co-designing of services and activities in others (Her Majesty's Prison & Probation Service, 2018; Treacy et al., 2019). It was suggested that this collaborative way of working should be supported by an information sharing protocol, clear definitions of staff and peer supporter roles and responsibilities, and training (Brooke & Jackson, 2019; Dillon et al., 2019; du Toit & Ng, 2022; HMP Littlehey, 2016; Turner, 2018). It was reported that there had been a lack of communication and coordination of this process in some prisons which had a negative impact on all involved (Brooke & Rybacka, 2020; Forsyth et al., 2020; Moll, 2013; Prisons and Probation Ombudsman, 2016).

It was also suggested that the prisons collaborate with healthcare, hospice and dementia specialists in the community and with external charitable organisations (Brooke et al., 2018; Brown, 2014; Cipriani et al., 2017; du Toit & Ng, 2022; Gaston, 2018; Gaston & Axford, 2018; Goulding, 2013; HMP Hull, 2015; HMP Littlehey, 2016; Her Majesty's Prison & Probation Service, 2018; Moll, 2013; Peacock, 2019; Prisons and Probation Ombudsman, 2016; Sindano & Swapp, 2019; Tilsed, 2019; Treacy et al., 2019; Welsh Government and Ministry of Justice, 2011; Williams, 2014). In addition, inter-prison networks were recommended to be developed to share good practice across prisons (Dementia Action Alliance, 2017; Moll, 2013; Peacock et al., 2019; Prisons and Probation Ombudsman, 2016).

##### Information-sharing

A number of papers (*n* = 7) recommended the need for a clear information sharing protocol regarding the assessment and support of PLiPWD (Brooke et al., 2018; Dillon et al., 2019; Department of Health, 2007; Goulding, 2013; Moll, 2013; Tilsed, 2019; Welsh Government and Ministry of Justice, 2011), or a register (Forsyth et al., 2020). Particular attention to the interface between healthcare and prison staff and peer supporters was suggested, where it has been reported that privacy regulations have sometimes prevented contributions to collateral histories (Feczko, 2014) and the sharing of care plans, impairing their ability to offer appropriate support (Inspector of Custodial Services, 2015). Also, it may be against the wishes of the person with dementia, and informed consent should be sought (Forsyth et al., 2020; Moll, 2013). This lack of information can have a detrimental effect on a person’s health and wellbeing (Brown, 2014, 2016; Feczko, 2014; Inspector of Custodial Services, 2015), and so discussion of this was highlighted as important, particularly where the safety of the person or others were concerned (National Institute for Health and Care Excellence, 2017). A care plan which gives only very basic information to staff and peer supporters was used in a couple of prisons (Goulding, 2013; Williams, 2014).

There also appeared to be variance with respect to whether healthcare staff disclose a dementia diagnosis to the person diagnosed with dementia. A couple of prisons’ policy was to share a diagnosis and involve family in doing so (Maschi et al., 2012; Welsh Government and Ministry of Justice, 2011; Wilson & Barboza, 2010), however, in one prison disclosed if a person was judged to be able to cope with it, and another only disclosed if asked (Brown, 2016). The importance of disclosure to family allowing them to contribute to assessments, planning and support was also emphasised in some papers (Brown, 2016; Dillon et al., 2019; National Institute for Health and Care Excellence, 2017; Welsh Government and Ministry of Justice, 2011).

## Discussion

This review has explored the literature regarding all parts of the custodial process and its impact on people living in prison with cognitive impairment and dementia, which includes: reception, assessment, allocation, training, policy, healthcare, accommodation, adaptation, routine, access to family and external agencies, transfer and resettlement. We found evidence that problems had been identified in each of these parts of the process. We also identified a number of cross-cutting themes which interacted with the issues identified across the prison journey including: principles or philosophy regarding care; capacity; resources; considerations of risk; scope for choice; peoples’ protected characteristics; collaboration; and, information sharing. Broadly, our findings were similar to those found in previous reviews, regarding the problems with the prison process identified, and the lack of robust outcomes, and policy guidance regarding PLiPWD (Brooke and Rybacka, 2020; Peacock et al., 2019).

The aim of this review was to identify areas of good practice and for recommendations that could inform the development of prison dementia care pathways. There is a considerable breadth to the findings, but the main recommendations that have arisen from the review are:

- To screen prisoners for cognitive difficulties at reception, from either 50 or 55 years
- An initial older-person specific health and social care assessment, post-screening – from either 50 or 55 years, and repeated (from 3 – 12 months)
- A spectrum of healthcare to be delivered including preventative, long-term and palliative care, with continuity of care upon release, and in tandem with social care
- Mixed views about appropriate accommodation, but it needs to run along a continuum from independent living to 24-h care, with decisions possibly made after health assessments
- Environments need to be made more older-person or dementia friendly, using checklists available, and with the voluntary sector as potential partners
- A need for prison staff training on dementia, and further training for healthcare staff
- The use of peer supporters was broadly reported positively, and were seemingly frequently used. However, there needs to be adequate training and support, and not to be used to do the work that is the statutory duty of health and social care staff
- Equal access to activities and services, especially programmes which help people move through the system (such as offending behaviour), as well as opportunities to earn additional monies, and that provide structure and routine on wings
- The maintenance of family links, and for families to be supported, are important for PLiPWD, and may be particularly so on release and resettlement
- Prisons may also need to work with external care agencies to ensure placements upon release, or alternative specialist care facilities may need to be created

The main barriers to implementing these recommendations are a lack of policy or guidance at local, regional and national levels to support staff in working with PLiPWD, and also the lack of budget and resources available. The latter would also include infrastructure issues, such that a number of prisons are not appropriate for people living with dementia, and could be expensive to modify to become so, coupled with a lack of currently available alternative facilities for PLiPWD to be released to in the community. The lack of use of compassionate release is also an issue here, including during the COVID-19 pandemic, with only 54 people released (Halliday & Hewson, 2022). Lastly, the roles that each professional and peer group had regarding PLiPWD needed clarification in some prisons, including some resolution of the ‘clash’ of philosophies (control v care) underpinning this.

In terms of ‘solutions’, multiple organisations have advocated for years for the need for national policy to assist prisons with older people in prison, including those living with dementia (Cornish et al., 2016; HM Inspectorate of Prisons, 2004, 2019; Prisons & Probation Ombudsman, 2016, 2017). This was eventually accepted and commissioned by the UK government, although it has not been released as yet (Justice Committee, 2020). It has also been suggested that at a more local level, existing policies could be adapted to be more appropriate for PLiPWD – such as restraint policies for frail prisoners, and disciplinary procedures which reflect the impact that dementia may have on behaviour (Department of Health, 2007; Treacy et al., 2019). Considerations around capacity and consent would need to be weaved in, as well as a focus on the intersection with other protected characteristics. These adaptations would also need to extend to services and activities to ensure that people have equal access and opportunities. A number of reports highlighted the contribution that greater collaboration with partners in external health and social care teams could have, as well as partnerships with the voluntary sector. These could potentially assist in multiple areas including training staff and peer supporters, providing activities, assisting release preparation, at a relatively low cost, to high benefit. There were some recommendations that prisons adopt a whole-prison approach to dementia that focuses on being person-centred, health and human rights focused that may help to ameliorate some differences in philosophical approach between various staff and peer groups in prisons.

A number of potential areas for future research were also indicated by the literature, which would also support the development of prison pathways. These would include: (i) induction to prison, and (ii) release and resettlement from prison, which are important beginning and end-points, but which are under-researched; (iii) the validation of a screening tool for use in prisons, and the development or adaptation of prison-specific health and social care assessments; (iv) the interaction of protected characteristics and dementia, and the need for more culturally and gender aware pathways; (v) the paucity of research conducted in low and middle-income countries, that needs to be addressed; (vi) dementia and age-related stigma in prisons; and (vii) evaluations of all elements of the prison pathway for PLiPWD to undertaken including training, the role of peer supporters, and targeted programmes.

## Strengths and limitations of the review

One key strength of this review is its comprehensiveness, particularly as it includes much grey literature. Given the lack of robust evaluation in this area, it was felt that this was necessary to represent the volume of work that has nonetheless taken place. There are, however, a number of limitations of this review. Firstly, despite the use of broad search terms, there may be the possibility that some relevant research was missed, either because of deficiencies in our searches or because of publication bias. Additionally, whilst there are twenty-two guidance and inspection documents included in this review, it is possible that some grey literature might also remain unidentified, particularly outside of the UK where the review was undertaken. Secondly, this review may be subject to a selection bias, as the yielded search results might have included literature that were excluded but which may have indirectly impacted upon the care pathways elements explored in the review. There is also a language bias, and whilst this may reflect the languages spoken by the review team members, it is also reflective of the “northern epistemic hegemony” (Aas, 2012), that also may have resulted in the review being largely populated by papers from high income countries. Thirdly, no formal assessment of study quality was undertaken. This is in keeping with scoping review methodology which focuses on breadth, but is nonetheless an important shortcoming inherent in scoping reviews more generally (Arksey & O’Malley, 2005).

## Conclusion

We have completed the most comprehensive review of the literature on PLiPWD in prisons to date that we have found, including a synthesis of the extensive grey literature, and found important gaps in the literature. Our review includes a mixture of academic research, policy and position papers which identified an increasing number of prisoners with dementia or cognitive impairment as an issue, but there were more limited descriptions of what should be done, and even less describing implementation of these. Most of the literature came from developed nations where extensive assessment and care services are in place for PWD in the community, although a key question is whether prison populations are given easy access to these existing services or whether bespoke services for prisoners are required. We suggest this literature now needs to be drawn together to inform interventions for PLiPWD in the criminal justice system which can be piloted and evaluated, and inform the development of robust dementia care pathways for prisons.

### Supplementary Information

**Additional file 1. **Preferred Reporting Items for Systematic reviews and Meta-Analyses extension for Scoping Reviews (PRISMA-ScR) Checklist.

**Additional file 2. Appendix 2:** Example search strategy.

## Data Availability

All data and materials used in this review are included in this article and its appendices.

## Footnotes

1 There is no standard cut-off age for older people living in prison, but it is typically set at least ten to fifteen years lower than the general population. People in prison are thought to age more rapidly due to both pre- and post-imprisonment chaotic lifestyles, substance misuse and less healthcare access and use, as well as the ‘pains of imprisonment’. See Williams et al., (2012) for further discussion.
