# Supplementary material for: Dementia care pathways in prisons – a comprehensive scoping review
Source: Health Justice. 2024 Jan 20;12:2. doi: 10.1186/s40352-023-00252-7 (PMC10799435; doi:10.1186/s40352-023-00252-7)
Supplement: Supplementary file 2 — Additional file 2. Appendix 2: Example search strategy. [file 40352_2023_252_MOESM2_ESM.doc]

**Appendix 2: Example search strategy**

Database: Ovid MEDLINE(R) and Epub Ahead of Print, In-Process & Other Non-Indexed Citations, Daily and Versions(R) <1946 to May 31, 2019>

Search Strategy:

--------------------------------------------------------------------------------

1 Dementia.mp. or Dementia/ (116335)

2 Alzheimer Disease/ or alzheimer*.mp. (147485)

3 1 or 2 (220022)

4 Prisons/ or prison*.mp. (27429)

5 jail*.mp. (3287)

6 gaol*.mp. (111)

7 correctional*.mp. (3339)

8 penal.mp. (1649)

9 penitentiary.mp. (514)

10 incarcerate.mp. (41)

11 incarceration.mp. (5430)

12 4 or 5 or 6 or 7 or 8 or 9 or 10 or 11 (34340)

13 3 and 12 (95)

***************************
